# Deciphering the tumor microenvironment and role of immunotherapy in diffuse midline glioma: A scoping review

**DOI:** 10.1093/neuonc/noag014

**Published:** 2026-02-02

**Authors:** Christian K Ramsoomair, Felipe Sarmiento, Deryn Ramsoomair, Manav Daftari, Jiasen He, Benjamin Glazer, Michelle Monje, Danny Reinberg, Ashish H Shah

**Affiliations:** Department of Neurosurgery, University of Miami, Miller School of Medicine, Miami, FL, USA; Sylvester Comprehensive Cancer Center, University of Miami, Miller School of Medicine, Miami, FL, USA; Medical Scientist Training Program, University of Miami, Miller School of Medicine, Miami, FL, USA; Department of Neurosurgery, University of Miami, Miller School of Medicine, Miami, FL, USA; Sylvester Comprehensive Cancer Center, University of Miami, Miller School of Medicine, Miami, FL, USA; Department of Neurosurgery, University of Miami, Miller School of Medicine, Miami, FL, USA; Sylvester Comprehensive Cancer Center, University of Miami, Miller School of Medicine, Miami, FL, USA; Department of Neurosurgery, University of Miami, Miller School of Medicine, Miami, FL, USA; Sylvester Comprehensive Cancer Center, University of Miami, Miller School of Medicine, Miami, FL, USA; Department of Pediatrics, The University of Texas MD Anderson Cancer Center, Houston, TX, USA (J.H.); Department of Neurosurgery, University of Miami, Miller School of Medicine, Miami, FL, USA; Sylvester Comprehensive Cancer Center, University of Miami, Miller School of Medicine, Miami, FL, USA; Department of Neurology and Neurological Sciences, Stanford University, Stanford, CA, USA (M.M.); Howard Hughes Medical Institute, Stanford University, Stanford, CA, USA; Howard Hughes Medical Institute, University of Miami, Miller School of Medicine, Miami, FL, USA; Department of Human Genetics, University of Miami, Miller School of Medicine, Miami, FL, USA; Department of Neurosurgery, University of Miami, Miller School of Medicine, Miami, FL, USA; Sylvester Comprehensive Cancer Center, University of Miami, Miller School of Medicine, Miami, FL, USA

## Abstract

Diffuse midline glioma, H3 K27-altered, formerly known as diffuse intrinsic pontine glioma, (DIPG/DMG) is the most aggressive form of pediatric brain malignancy, with <10% 2-year overall survival after standard of care. The limited success of traditional immune checkpoint inhibitors in pediatric high-grade gliomas, including DMG, has highlighted the urgent need to re-examine the tumor’s intrinsic and microenvironmental barriers to immunotherapy. Advances in molecular and spatial profiling have revealed the profound intratumoral heterogeneity, lineage plasticity, and complex immunosuppressive tumor microenvironment characteristic of DMG, which are shaped by diverse myeloid populations, neuronal integration, and spatially distinct tumor niches. These insights are informing the development of non-traditional immunotherapeutic approaches, including alternative checkpoint blockade, chimeric antigen receptor T cells, and viro-immunotherapy strategies, which aim to overcome DMG’s unique immune escape mechanisms. We also outline key translational challenges and future directions necessary to accelerate progress, including the refinement of preclinical models, optimization of central nervous system (CNS)-specific immunotherapy delivery, and the integration of patient-derived data into streamlined, collaborative clinical trial platforms.

Tumors of the central nervous system (CNS) are the most common solid malignancy in children as well as the predominant pediatric cause of cancer-associated mortality. The most aggressive CNS neoplasms in children are classified as pediatric high-grade gliomas (pHGGs), which initiate from oligodendroglial lineage precursors in the developing CNS. Diffuse midline glioma (DMG) is a subclassification of pHGGs that originates along the midline structures of the brain (Figure 1A).^1^^,^^2^ When located in the pons, it is also known as diffuse intrinsic pontine glioma (DIPG). Clinicians may also still refer to a tumor as DIPG if it does not meet the molecular requirements to be DMG, or if they are not biopsied. Diffuse midline gliomas account for approximately 20% of all pediatric CNS cancer diagnoses, which is nearly 350 new cases each year in the United States alone. The overall survival (OS) of patients diagnosed with DMG is extremely poor at 9-11 months with a 2-year survival of less than 10%.^3^^,^^4^

**Figure 1.**
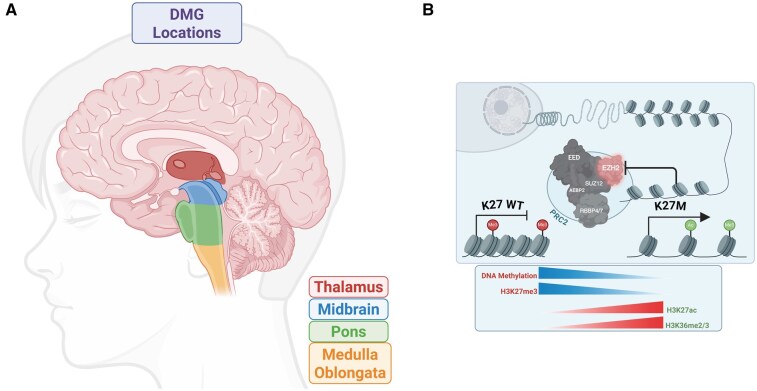
Anatomical and molecular characteristics of DMG. (A) DMG predominantly arises in the midline structures of the brain and brainstem. (B) The K27M substitution in the tail of H3 leads to a global epigenetic dysregulation. Created in BioRender. Shah, A. (2025) https://BioRender.com/0fuci4p

## Classification, Epigenetics, and Molecular Subtypes of DMG

**Figure 2.**
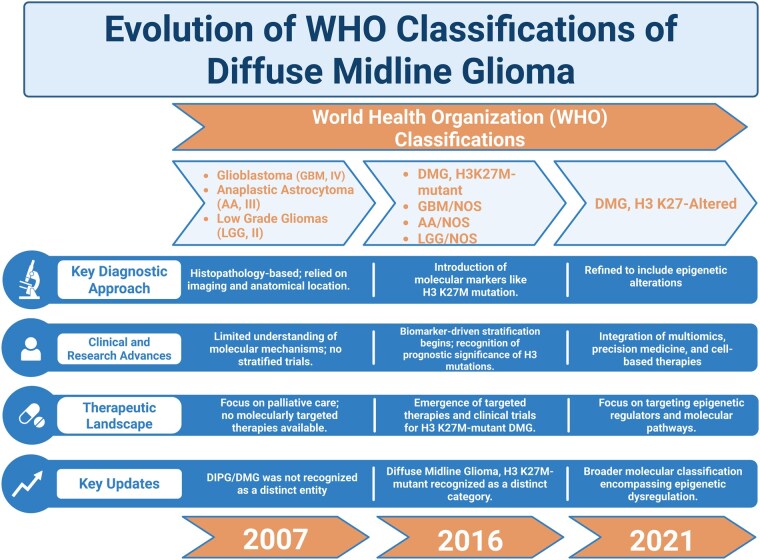
Evolution of the WHO Classification of DMG. In 2007, DIPG/DMG was not recognized as a distinct entity. These tumors were histologically classified as glioblastomas (GBM, grade IV), anaplastic astrocytomas (AA, grade III), low-grade gliomas (primarily diffuse astrocytomas, LGG-DA, or grade II). By 2016, most cases were classified as DMG, H3K27M-mutant tumors. However, H3K27 WT DMGs remained unclassified. The 2021 WHO CNS Classification integrated all pediatric DMG cases with H3K27 alterations as including the loss of H3 K27 trimethylation. Abbreviations: AA, anaplastic astrocytoma; DA; diffuse astrocytoma; DIPG, diffuse intrinsic pontine glioma; DMG, diffuse midline glioma; GBM, glioblastoma; IDHwt, IDH wild-type; LGG, low-grade glioma; NOS, not otherwise specified. Created in BioRender. Shah, A. (2025) https://BioRender.com/qjqriay

The World Health Organization (WHO) classification and overall approach toward DMG has been revised several times in recent years as summarized in Figure 2. As of the fifth edition of the WHO’s Classification of CNS Tumors in 2021, DMG is designated as “H3 K27-altered,” (alteration at lysine 27 of histone 3) emphasizing that global hypomethylation of H3K27 is a hallmark feature of DMG.^5^^,^^6^ Mechanistically, this is driven by either recurring somatic mutations in H3 genes, including *HIST1H3B/C* (H3.1K27M) or H3F3A (H3.3K27M) in which the lysine 27 residue is substituted with methionine, or overexpression of the Enhancer of Zest Homologs Inhibitory Protein (EZHIP) in patients with H3 wildtype. These alterations drive oncogenic transcriptional programs for which few effective treatments currently exist. It is important to note that the H3.1 and H3.3 histone variants are mutually exclusive. H3.1K27M accounts for approximately 12%-19% of DMG cases, with a median OS rate of 15 months, while 65% of cases are characterized as H3.3K27M, with a median OS of 9 months.^7^ Wildtype H3 is identified in 10%-15% of cases, with a median OS of 15 months.^8^  Table S1 summarizes the implications for immunotherapy response by subtype, with a more in-depth discussion later in this review.

A defining characteristic of DMG is its global hypomethylation. Driven by recurring somatic mutations in H3 genes or EZHIP overexpression, these alterations inhibit the catalysis of repressive epigenetic marks on K27 of histone H3 by the Polycomb repressive complex 2 (PRC2).^9-12^ In normal tissues, a myriad of marks decorate K27 on histone H3. These include either acetyl (H3K27ac), monomethyl (H3K27me1), dimethyl (H3K27me2), or trimethyl (H3K27me3). While H3K27ac and H3K27me1 are associated with active transcription, the deposition of H3K27me2 and H3K27me3 are catalyzed by PRC2 and associated with transcriptional repression. It is important to highlight that H3K27M has been shown to lead to global disruptions of not only H3K27me3^9^^,^^10^^,^^13–16^ but also DNA methylation^13^^,^^16^ and H3K9me3^17^ as well as increased levels of marks associated with active transcription such as H3K4me3,^18^ H3K36me2,^14^^,^^19^ and H3K27ac^2^^,^^20^ (Figure 1B). Interestingly, the H3K27M substitution impacts the methylation of not only the mutant allele but all H3 alleles.^9^^,^^10^^,^^12^ Thus, even though only a fraction of total histone H3.1 and H3.3 harbor the K27M mutation, a global reduction of H3K27me2/H3K27me3 persists in each DMG cell.^10^ This dominant negative phenotype of H3K27M makes it analogous to EZHIP overexpression where the global loss of transcriptional silencing is strongly associated with DMG gliomagenesis.

## Challenges from Targeting Genomic Aberrations and Affected Pathways in DMG

**Figure 3.**
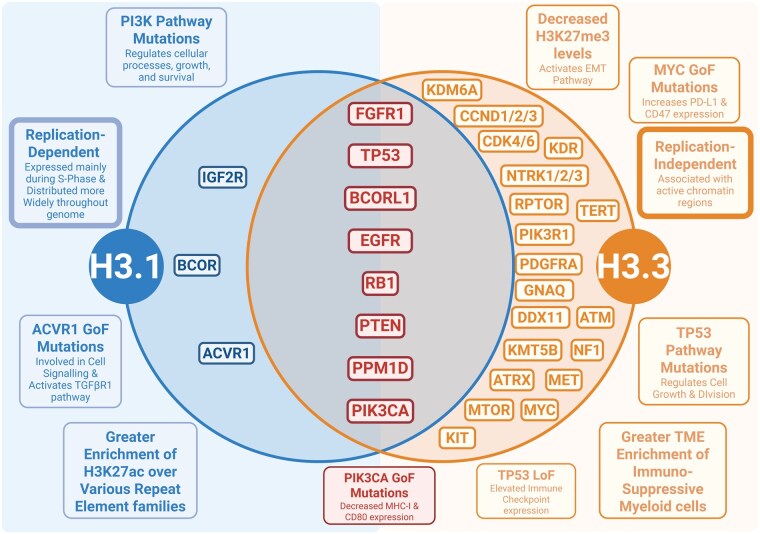
Somatic mutation profiling of H3.1K27M versus H3.327M DMGs. A venn diagram summarizing common somatic mutations between DMGs driven by either H3.1K27M (left, blue) and H3.3K27M (right, orange) or both (middle, red). Text boxes highlight effects specific to the respective driver mutation. Created in BioRender. Shah, A. (2025) https://BioRender.com/b7iypf2

Diffuse midline glioma has a low mutational rate compared to other cancers, but is frequently associated with mutations in genes involved in cell cycle regulation (*TP53*, *PPMID*), chromatin remodeling (*ATRX*), and growth factors (*ACVR1*).^21^ Profiling of the common somatic mutations between the H3K27M mutant subtypes are summarized in Figure 3.^22^ See **Section S1** for a detailed discussion of subtype-specific co-mutations and evolutionary drivers (including H3 variant partnerships).

Diffuse midline glioma tumors exhibit high levels of both inter- and intra-tumoral clonal heterogeneity, with multiple somatic subclones co-existing in the spatial and temporal dimensions.^23^^,^^24^ High-throughput drug screening campaigns with molecular analysis have been employed to assess drug sensitivities in a plethora of DMG models, which aim to correlate genomic influences with potential treatment strategies.^25^^,^^26^ Subclones are crucial to tumorigenesis, enhancing proliferation, invasion, and oncogenic signaling, particularly in response to CNS-active therapies.^22^ Thus, given the complexity of the clonal cancer genome, a one-size-fits-all targeted approach is likely destined for failure as tumors typically harbor multiple mutations with unclear significance, which may complicate the identification of oncogenic drivers.

Transplanting or co-culturing DMG subclones with less mobile colonies has been shown to augment invasiveness, which suggests a cooperative role in tumor cell dissemination.^24^ While targeting specific subclone mutations may provide temporary benefits, the genomic diversity of subclones will ultimately limit the long-term efficacy of targeted approaches.^27^ Furthermore, the tumor microenvironment (TME) and non-genomic factors such as corticosteroid therapy, growth factors, and stress hormones have been found to contribute to increased invasiveness and intertumoral heterogeneity, complicating the treatment efforts. Moving forward, it is critical that therapeutic regimens for DMG consider not only the characteristics of the tumor itself but also the microenvironment that encapsulates and protects it.

## The Tumor (Immune) Microenvironment

**Figure 4.**
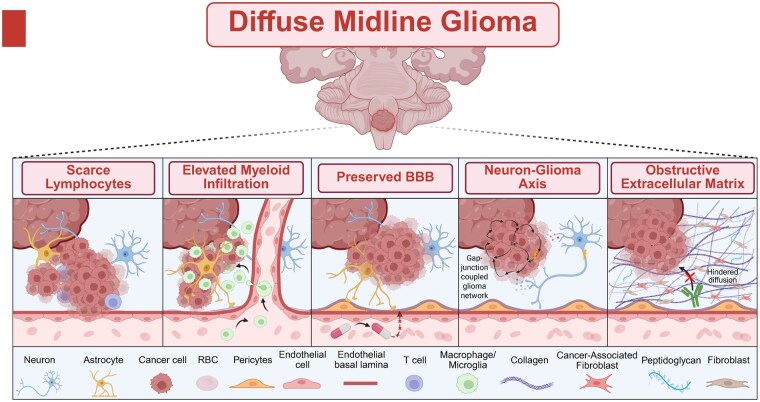
The Hallmarks of the DMG TME. Cartoon schematic showcases the 3 major characteristics of the tumor microenvironment (TME) of diffuse midline glioma (DMG): (1) Scarce lymphocytic infiltration, (2) increased myeloid cell populations, (3) an intact BBB, and (4) neuron-glioma interactions. Created in BioRender. Shah, A. (2025) https://BioRender.com/fc7bo2f

While the heterogeneous nature of HGGs like DMGs has stagnated improvements in targeted therapies for decades, landmark studies on the TME of DMGs over the past couple of years (secondary to increased access to biopsy samples) have revolutionized our view and reinvigorated the field’s interest in the potential for immunotherapies (Figure 4).

Historically, the CNS has been described as immunologically privileged tissue containing little to no immune cell infiltration. However, this has been disproven by extensive evidence demonstrating immune cell transfer between the CNS and the periphery via the glymphatic system, a glia-based lymphatic drainage system, the choroid plexus, meninges, and perivascular spaces.^28-33^ Studies have shown adaptive immune trafficking as well as peripheral immune cell-mediated inflammatory responses in the CNS that have been shown to even regulate social behaviors such as stress.^34-37^ Amongst the many specialized immune cell populations in the CNS, microglia, specialized tissue-resident macrophages, are well-characterized to be the primary resident immune cell of the brain with critical functions such as supporting brain development,^38^ neural circuit refinement,^39^^,^^40^ and immune surveillance.^38^^,^^41^

The midline structures of the brain and brainstem, as eloquent areas, are vital for coordinating motor functions like swallowing and essential bodily functions such as cardiac and respiratory control. Thus, it is critical to strike a balance between protection against pathogens and immune-mediated neuroinflammation.^42^ For this reason, control of peritumoral inflammation with dexamethasone (corticosteroid) is commonly prescribed to children with DMG. However, dexamethasone has been shown to induce T-cell exhaustion (upregulation of Immune Checkpoint ligand cytotoxic T lymphocyte-associated antigen-4 [CTLA-4]), and thus has been suggested to exacerbate the immunologically cold-DMG TME.^42^^,^^43^

Studies that have analyzed the TME of DMG have found a paucity of lymphocytes and chemokines compared to normal brain tissue.^44^ Moreover, the expression of CD3 (T-cell specific marker) as well as expression of immune activating cytokines and chemokines in DMG is substantially lower than both adult glioblastoma and other pediatric gliomas.^45^ Corroborating this finding, transcriptomic and immunohistochemistry (IHC) analysis of autopsy samples from DMG patients have found that the majority of DMG tumors have little to no infiltrating CD8+ cells.^44^^,^^46^ Remarkably, IBA1 (a marker for macrophages/microglia) has been shown to be markedly upregulated in primary DMG tissue.^45^ This has led many to speculate that these myeloid-derived cells and microglia may have a critical role in DMG immunosuppression, but have not been characterized until recently in the studies discussed below.

### Recent Advancements From Single-Cell, Spatial Analyses, and Preclinical Models

Single-cell and spatial analyses have been paramount in the characterization of the DMG TME. However, it is important to note these technologies were first employed to determine the tumor cell populations themselves. Though debated,^47^ many studies support an early oligodendroglial precursor as the predominant cell of origin for DMG,^2^^,^^48–55^ including histologic analysis of the developing human brainstem identify pre-Oligodendrocyte precursor cells (OPCs) in the pons at the time and place where DIPG typically arises.^48^ Super-enhancer profiling also indicates that OPC/pre-OPC chromatin states closely mirror DMG, compared to neural precursors,^2^^,^^53^ and single-cell RNA sequencing shows malignant DMG populations are dominated by proliferative OPC-like cells where H3K27M impedes terminal glial differentiation.^56^^,^^57^ A landmark multi-omic study of DMG throughout various age groups found a striking absence of neuronal-lineage tumor cells in DMG and proposed that K27M enforces a glial/OPC-like program irrespective of age or site.^55^ Of note, previous work has suggested that these “glioma stem cells” play a fundamental role in the suppression of both the innate and adaptive immune system by immunosuppressive cytokine secretion.^58^^,^^59^

The Filbin group observed that glioma-associated myeloid cells (GAMs) comprise the largest population of nonmalignant cells within DMG tumors,^55^ consistent with previous histological studies demonstrating GAMs as the predominant immune cell present in DMGs.^45^ Though overall GAM proportions were consistent between tumors from adult and children, pediatric DMGs displayed higher rates for brain-resident microglia while adult DMGs were enriched with monocyte-derived macrophages. The authors highlight evidence that GAMs secrete ligands (such as Oncostatin M [OSM]), contribute to the mesenchymal cell state in adult glioblastoma, and were also found to promote MES-like tumor cell states in H3-K27M DMG-associated GAMs as age increases.^60–62^ This marked one of the first instances where microenvironmental factors were found to distinctly shape tumor cellular states in DMG, which has propelled further investigation into the role of the myeloid population in H3K27M tumors.

In 2024, a series of breakthrough papers shed light on the TME of DMG, specifically the myeloid-derived subpopulations, which has critical clinical implications for future immunotherapies. While we provide a summary below, please refer to our expanded discussion in **Section S2** for a more thorough analysis.

Using single-cell RNA sequencing (scRNA‑seq) plus imaging mass cytometry, Andrade et al. reported DMGs contain myeloid‑dominant infiltrates with modest T‑cell presence, and K27M‑derived microglia have immunosuppressive signatures and high immune checkpoint expression.^63^ Thus, highlighting the potential role of myeloid cells in promoting the immunosuppressive TME. Serial engraftments in a syngeneic K27M model accelerated growth and progressively skewed the TME toward myeloid dominance. Moreover, while monotherapy targeting microglia (CSF1R) or anti‑PD‑1 was ineffective, combinatorial approaches increased CD3⁺ infiltration and extended survival, implicating myeloid modulation as a prerequisite for T‑cell recruitment.^63^ Complementary work from Ross and colleagues show that H3.3K27M DMGs are enriched for disease‑associated microglia/monocyte-derived macrophage (MDMs) that downregulate inflammatory signaling and facilitate immune escape. Similarly, they found that targeting this microglia state (dual CCR1/CCR5 inhibition) reduced myeloid recruitment, increased CD8⁺ infiltration, and prolonged survival to an extent comparable with radiotherapy, without altering circulating myeloid counts.^64^ Collectively, these studies highlight the importance of co-targeting microglia/myeloid populations with traditional immunotherapeutic approaches (like anti-Programmed cell death protein-1 [PD-1]) to overcome the immunosuppressive DMG TME.

Despite the DMG TME being described as immunosuppressive, a large‑scale immune‑oncology profiling in a cohort of 1382 total pediatric brain tumors demonstrated an unexpectedly high tumor inflammation signature (TIS) score in DMG relative to non‑tumor brain and hemispheric HGG. Immunohistochemistry confirmed high expression of several immune checkpoints on a subset of DMG samples including those with currently available FDA-approved drugs (PD-1, programmed cell death ligand protein-1 [PD-L1]) and drugs under development (LAG3, TIGIT).^65^ They also assessed the utility of TIS compared to tumor mutational burden (TMB) for its use as a biomarker for predicting immunotherapy treatment response. The best outcomes occurred in recurrent HGG patients with both high TIS and TMB, several achieving over 3 years of progression-free survival. This data suggests that a subset of DMG patients may be candidates for radiation- and chemotherapy-free regimens given their robust immunotherapy response.^65^^,^^66^ Stratification in the neoadjuvant setting, utilizing biomarkers such as TIS/TMB, holds tremendous promise to change how we view the DMG TME as an monolith entity.

Although large-scale transcriptomic datasets have transformed the field and are increasingly clinically relevant, recent spatial multi-omic analyses by Damodharan et al. caution against relying solely on RNA reads for TME profiling and target selection. Digital spatial profiling revealed limited RNA-protein concordance in DMG, with notably poor alignment for clinically relevant immune checkpoints (PD-L1, CTLA-4) and Epidermal growth factor receptor (EGFR). These findings underscore the importance of incorporating proteomics into discovery pipelines and early-phase trial design.^67^^,^^68^

Collectively, these advances delineate a DMG ecosystem dominated by OPC‑like tumor states and immunosuppressive myeloid programs, identify biomarker frameworks (TIS/TMB) that may enrich for immunotherapy benefit, and point to combination strategies that pair myeloid‑targeting agents with checkpoint blockade to overcome resistance.^63-66^

## DIPG/DMG Integration With Neural Networks

Although the majority of studies in DMG focus on immune-resident population, few studies have suggested the critical role of neurons in DMG immunomodulation. Collectively, cancer neuroscience reframes DMG as a malignancy that is embedded within and sculpted by neural circuits, rather than a mass merely residing in the CNS. Building on developmental neurobiology, recent work from multiple groups shows that DMG co‑opts activity‑regulated programs to drive tumor intrinsic proliferation, invasion, and therapeutic resistance via secreted factors, bona fide synapses, neuromodulatory inputs, and coordinated immune crosstalk.^69^^,^^70^ This triangular interaction among neurons, glioma cells, and immune cells provides a mechanistic framework for why conventional therapies underperform and how circuit‑level interventions might potentiate immunotherapies.^71^^,^^72^ See our expanded discussion in **Section S3**.

During development, OPCs, a cell state closely linked to DMG, are regulated by neuronal activity,^73^ and the landmark study by Venkatash et al. illuminated that tumors exploit this relationship.^74^ They identified neuroligin‑3 (NLGN3) as an activity‑regulated, secreted factor that accelerates glioma growth. Neuroligin‑3 initiates a feed‑forward loop in tumor cells, engaging PI3K-mTOR and elevating oncogenic programs (PDGFA, TTYH1) across multiple glioma subtypes, including DMG. Follow‑up work discerned key mechanistic sources and consequences of NLGN3 in the glioma-context.^75^ In particular, the sheddase ADAM10 was identified as the mediator of NLGN3 release. Further, ADAM10 inhibition was shown to reduce tumor burden in DIPG models, highlighting a tractable extracellular target and demonstrating neuronal paracrine signaling as a major mitogenic driver in DMG.

Beyond secreted signals, DMG cells were also found to electrically integrate into neural networks. Venkatesh and colleagues pioneered this discovery that tumor cells form excitatory neuron-to-glioma synapses mediated by calcium-permeable α-amino-3-hydroxy-5-methyl-4-isoxazole propionic acid (AMPA) receptors, while also exhibiting prolonged, non-synaptic depolarizations driven by activity-dependent extracellular potassium that propagate through gap-junction-coupled glioma networks.^76^ These inputs increase proliferation, and pharmacologic blockade of AMPA currents or gap‑junction signaling significantly reduced tumor growth in vivo.^74^^,^^76^ Importantly, independent work in glioblastoma corroborated glutamatergic synapses to glioma cells, underscoring the generality of neuron-glioma electrochemical communication.^77^

As CNS regulation of cancer became increasingly appreciated, more complex questions, including adaptive plasticity, arose. In pediatric glioma models, Taylor and colleagues revealed neuronal firing induces an adaptive response in malignant synapses via a brain-derived neurotrophic factor (BDNF)-TrkB-CAMKII axis. Diffuse midline glioma upregulates the BDNF receptor, TrkB, which allows for augmentation of excitatory currents, synapse number expansion, and growth acceleration. Genetic deletion or pharmacologic inhibition of TrkB weakens these malignant synapses and prolongs survival.^70^ These results reveal that gliomas not only passively receive neuronal input but actively reinforce and expand malignant synapses through activity-regulated plasticity mechanisms. Analogous to learning and memory processes in healthy brain circuits, gliomas hijack the BDNF-TrkB-AMPAR axis to sustain excitatory signaling that fuels their proliferation.^70^ The work highlights TrkB inhibition as a promising therapeutic strategy, particularly as clinically approved TRK inhibitors could be repurposed for non–NTRK fusion gliomas.

Interestingly, tumor-intrinsic epigenetic dysregulation further shapes how DMG integrates into neural circuits.^78^ In H3.1K27M DMG, Zhang and colleagues elucidate that dysregulated epigenetic mechanisms increase neuron-to-glioma synapses in DMG, specifically the CDH2-FOSL1 axis. Genetic depletion of this axis reduces active chromatin marks, downregulates synaptic programs, and diminishes neuron-induced proliferation, independent of canonical regulators (NLGN3, BDNF, and TRKB).^78^ Despite this epigenetic axis being highly targetable, different subtypes likely exhibit distinct epigenetic mechanisms to mediate these interactions, and thus future work is needed.

Most recently, studies have addressed a longstanding gap in the field by showing that DMG also exploits non-glutamatergic neurotransmission to its advantage. Secondary to NKCC1‑mediated chloride accumulation, GABAergic inputs become depolarizing (growth‑promoting) in DMG.^79^ In preclinical models, enhancing GABA signaling with benzodiazepines increases tumor growth, whereas IDH‑wild‑type hemispheric HGGs show minimal depolarizing GABA currents, highlighting an important clinical consideration for DMG patients.^79^^,^^80^ In parallel, neuromodulatory brainstem circuits have been found to exert region‑specific control. Cholinergic projections from the pedunculopontine nucleus (PPN) promote pontine DMG growth, and laterodorsal tegmentum inputs stimulate thalamic disease via muscarinic receptors (CHRM1/CHRM3). In DMG xenografts, targeted antagonism abolished this proliferative response.^81^

In summary, DMG integration into synaptic networks is pervasive and robust. Increased expression of synaptic proteins and enhanced neuronal signaling cooperate to drive glioma proliferation in a durable positive feedback loop. Ongoing work seeks to uncover the role of immune cells in this crosstalk, and if immunotherapy can combine with circuit‑level blockade (eg, ADAM10, AMPA, gap junctions, NKCC1, muscarinic or TrkB inhibition) to dismantle the neuronal drive while remodeling the tumor immune milieu.^70^^,^^75^^,^^76^^,^^79^^,^^81^^,^^82^

## Opportunities and Clinical Challenges with Immunotherapies in DMG to Date

### Immune Checkpoint Blockade

Immune Checkpoint Inhibitors (ICIs) function by enhancing anti-tumoral adaptive immunity by restoring cytotoxic T cell activity and reversing T-Cell exhaustion. Currently, the most common ICIs used clinically are antibodies against CTLA-4, PD-1, or PD-L1. These ICIs have had tremendous success in hematological cancers and other solid tumors, namely metastatic melanoma. Unfortunately, the preliminary use of ICIs in DMG has lacked efficacy.^83^ A list of the current ICI clinical trials for the treatment of DMG is summarized in Table S2.

In a retrospective cohort analysis, Kline and colleagues reported no significant difference in outcomes between patients treated with PD-1 blockade combined with re-radiation versus re-radiation alone.^84^ Nevertheless, new phase I and I/II clinical trials have been launched to evaluate the therapeutic potential of immune checkpoint inhibition in DMG. These trials include anti-PD1 therapies such as pembrolizumab (NCT02359565), pidilizumab (NCT01952769), and cemiplimab (NCT03690869), anti-PDL1 therapy with durvalumab (NCT02793466) and, most recently, a combination of anti-CTLA4 and anti-PD1 therapies with nivolumab and ipilimumab (NCT03130959), which did not improve survival in patients with newly diagnosed diffuse intrinsic pontine glioma following upfront irradiation relative to historical data.^42^^,^^85^ Though more recent evidence may imply a more complex relationship,^65^ it was previously postulated that this lack of response was due to the lack of PD-L1 expression on the DMG cell surface as well as the absence of T cells in the tumor microenvironment.^86^ As discussed in the previous section, combinatorial strategies to enhance immune checkpoint expression such as PD-L1, stimulate activating cytokines/chemokines, and target immunosuppressive myeloid populations in the DMG may be helpful in sensitizing tumors to ICI. In addition, recent attention has turned to less studied immune checkpoint pathways such as T cell immunoglobulin and mucin domain-containing protein 3 (TIM-3) and the CD47-signal retention protein alpha (SIRPa) pathways. These approaches may be more attractive in DMG since they also target (immunosuppressive) myeloid cells, which comprise the majority of the DMG TME.

In a recently published study, Ausejo-Mauleon and colleagues found that TIM-3 was highly expressed in both tumor cells and immune cells, namely microglia and macrophages, in DMG patients.^87^ Further, they demonstrated TIM-3 blockade in immunocompetent DMG orthotopic models prolonged survival, with 50% of long-term survivors being disease-free and acquiring immunological memory. This pre-clinical data offers strong support for future clinical trials with an anti-TIM-3 antibody against DMG as monotherapy or combined with other proven immunologic treatments, such as oncolytic viruses (OV) or Adoptive cell transfer (ACT).

Similarly, CD47, a well-studied anti-phagocytic ligand that is overexpressed in a variety of tumors,^88^ binds SIRPa from macrophage/dendritic cells (DC) to decrease their phagocytic capacity and promote tumor-mediated immune evasion.^88^ To counter this, Gholamin et al. developed a humanized antibody against CD47 that blocks this “don’t eat me” signal. Using patient-derived xenograft models of pediatric brain tumors, the antibody effectively inhibited CD47, killed tumor cells, and extended the animals’ survival without causing toxicity to normal tissues.^89^ These promising preclinical results prompted a clinical trial (NCT05169944), which seeks to employ the anti-CD47 antibody, magrolimab, against recurrent or progressive malignant brain tumors in both adults and children. Although this study excluded DMG patients, we suggest future studies consider including DMG patients, potentially as an exploratory arm, given the disproportionally high expression of CD47 in the DMG TME.

### Adoptive Cell Transfer

Adoptive cell transfer is an immunotherapy treatment wherein immune cells are isolated from a patient, modified, expanded *ex vivo*, and transferred back into the patient. Currently, T-cells genetically modified to express a chimeric antigen receptor (CAR-T) are the most popular form of ACT due to their remarkable clinical response rates in the context of hematologic malignancies, and have become the first FDA-approved ACT for refractory/relapsed B cell lineage acute lymphoblastic leukemia (B-ALL).^90^ Now, less than a decade later, 6 CAR-T therapies are commercially available for the treatment of various B cell lineage malignancies.^91^ This work has paved the way for their application in solid tumors, though achieving meaningful survival benefits continues to be a challenge. Given the “cold” immunological features of DMG, CAR-T cell therapy is touted by many as being the most promising immunotherapeutic approach with at least 10 CAR-T cell clinical trials for DMG, active as of December 2025. An exhaustive list of the current ACT clinical trials for the treatment of DMG are summarized in Table S3.

A CAR, or chimeric antigen receptor, is a fusion protein that incorporates 5 main components: an antigen-binding domain (typically composed of an antibody-derived single-chain variable fragment, or ScFv), a flexible hinge domain, a transmembrane domain, costimulatory domains (such as 4-1BB or CD28), and an intracellular signaling domain derived from the CD3ζ subunit of the T cell receptor (TCR) complex.^92^^,^^93^ Variations to CAR designs to include auxiliary genes, such as cytokine receptors, are also typical for enhancing their antigen recognition capabilities as well as signaling efficacy.

Effective T cell engagement and resultant tumor destruction relies upon the recognition of tumor-associated antigens (TAA) that are highly expressed on cancer cell surfaces in an MHC-independent manner. This triggers effector T cell signaling pathways, leading to the release of cytotoxic molecules such as perforin and granzyme B, along with proinflammatory cytokines like IL-2, IL-6, IFN-γ, and TNF-α.^92^ Thus, the selection of the TAA is critical to minimize on-tumor/off-target (OT/OT) toxicities. For example, an ideal TAA is homogeneously expressed on tumor cells but absent or minimally expressed in healthy tissues.^93^ Beyond CAR-T cell design, factors such as T cell subset phenotypes, macrophage populations, and circulating cytokines and chemokines also influence therapeutic efficacy. While early results from CAR-T cell therapies in DMG patients have shown promise for longer-term survival, intratumoral heterogeneity and the TME remain the primary therapeutic challenges.

Epidermal growth factor receptor is a well-studied therapeutic target linked with tumor progression and upregulation of a tumor-specific variant (EGFRvIII) in DMG.^94^^,^^95^ Numerous trials evaluating EGFR CAR-T therapy against HGGs have displayed safety and feasibility with some clinical efficacy. Two Phase 1 trials (NCT02209376, NCT01454596) utilizing EGFRvIII CAR-T cells against adults with HGGs have demonstrated tolerable responses with IV and intracerebroventricular (ICV) delivery, respectively.^96^^,^^97^ Although there were no evidence of OT/OT toxicities, neither trial demonstrated a clinically meaningful effect in patients. Currently, a Phase I trial (BrainChild-02, NCT03638167) is evaluating a CAR-T targeting EGFR806 in pediatric HGG patients but is excluding pontine gliomas. Thus, future trials are needed to show the efficacy of EGFR-targeting CARs against the classic DMG/DIPG tumors.

T-cells genetically modified to express a chimeric antigen receptor cell trials (NCT03500991, NCT02442297) targeting ERBB2 receptor tyrosine kinase (HER2) for CNS tumors at progression in both pediatric and adult settings have demonstrated safety and tolerability.^98^^,^^99^ Between these 2 trials, systemic, tumor cavity, and ventricular system infusion delivery were evaluated. Infusions via a CNS catheter showed evidence of local CNS immune activation, including high concentrations of CXCL10 and CCL2 in the cerebrospinal fluid.^98^ Whereas the trial with systemic administration found that of 16 evaluable patients (9 adults and 7 children), 1 had a partial response for more than 9 months, 7 had stable disease for 8 weeks to 29 months, and 8 progressed after T-cell infusion.^99^

Several CNS tumors, including DMG, highly express IL-13Rα2, which is targetable through a IL-13 cytokine-directed CAR.^92^^,^^100^ Interestingly, IL-13Rα2 is poorly expressed in healthy tissue, besides the testis, and thus has prompted clinical trials for DMG. Phase I clinical trials (NCT04510051) for pediatric brain tumor patients as well as (NCT02208362) for adults with glioblastoma are currently active. Although preliminary evidence supported the feasibility and tolerability of IL-13Rα2 directed CAR-Ts, disease recurrence occurred due to reduced IL-13Rα2 expression.^100^^,^^101^ We anticipate that combination treatment with ICIs may be the next step against DMG to parallel the Phase I trial (NCT04003649) for glioblastoma, which combines IL-13Rα2 CAR-Ts with the ICIs, nivolumab (PD1) and ipilimumab (CTLA4).

### Recent Landmark Successes for CAR-Ts in DMG

Screening the cell surface of patient-derived DMG cells, Mount and colleagues revealed that the now popular CAR-T antigen, disialoganglioside GD2, is highly and uniformly expressed on H3K27M cells and GD2-targeting CAR-T therapy has remarkable efficacy in preclinical models.^102^ Currently being investigated in 4 active clinical trials for DMG alone, GD2 has also been found to be expressed on several solid and CNS tumors while being minimally expressed in healthy tissue.^103^ In 2020, a Phase 1 clinical trial (NCT04196413) began recruiting to evaluate intravenous (IV) infusion of autologous GD2 CAR-T cell therapy, with the option for repeated intracerebroventricular dosing, in young adult and pediatric patients with H3K27M-mutant diffuse midline glioma. Impressively, preliminary data demonstrates radiographic and clinical benefit in 3 out of 4 patients, including significant tumor reductions and neurological improvements. Of note, the single non-responsive patient still exhibited an immune response of elevated levels of immunosuppressive cytokines, such as transforming growth factor β (TGF-β), in the cerebrospinal fluid (CSF). Importantly, ICV dosing was associated with improved or stabilized responses, reduced cytokine release syndrome (CRS), enhanced proinflammatory cytokine and chemokine levels in the CNS, and a decrease in immunosuppressive myeloid cells in the CSF compared to IV infusions. Tumor inflammation-related neurotoxicity (TIAN), likely due to the tumor’s midline location, caused transient neurological worsening in all 4 patients, with intratumoral edema contributing to temporary CSF obstruction and hydrocephalus, requiring intensive care management.

More recently, the final results of this phase I trial was presented by Monje et al. for NCT04196413, which assessed the feasibility and safety of intravenous and intracranial GD2-CAR-T cells for H3K27M+ diffuse midline gliomas.^104^ Reported adverse events included CRS and TIAN, which were effectively managed through intensive monitoring and comprehensive supportive care. Notably, ICV administration was associated with reduced systemic toxicity, emphasizing its potential as a targeted approach for gliomas while minimizing systemic immune activation. Promising clinical outcomes were observed, including substantial tumor volume reductions in multiple patients, with one patient achieving a complete response that has persisted for over 30 months. Clinical neurological improvements were generally consistent with radiographic findings, although some discordance between imaging outcomes and clinical metrics was noted, highlighting the complexity of response assessment in DMGs. Key mechanistic insights demonstrated the prolonged persistence of GD2-CAR-T cells in CSF and peripheral blood for over 500 days in select patients. This persistence was coupled with reductions in tumor-specific DNA markers and correlated with elevated cytokine levels in both CSF and blood, further supporting evidence of robust, on-target antitumor activity.

In 2024, results were published from NCT04099797, a Phase 1 trial assessing modified GD2-directed CAR-T cells with a constitutively active interleukin (IL)-7 receptor (C7R-GD2.CARTs).^105^ In an effort to improve expansion and cytotoxicity of CAR-T cells while avoiding toxicities associated with recombinant cytokines, this group at Texas Children’s Cancer Center engineered a constitutively active C7R to safely augment antitumor activity of CAR-T cells against glioblastoma (GBM) xenografts.^106^ Inspired by the early successes of the GD2 CAR-T against DMG, the authors combined their technology to assess the safety and efficacy of the C7R-GD2.CARTs against DMG and other recurrent GD2-expressing pediatric CNS tumors.^105^ Among patients treated with C7R-GD2.CART cells, partial responses were reported in 2 out of 7 patients. Seven out of 8 developed grade 1 TIAN and 6 out of 8 CRS, but all events were controlled with anti-cytokine agents. Of note, 7 out of 8 patients received multiple cycles and exhibited transient neurological and radiographic improvement with higher levels of granzyme B and interferon-γ expression in peripheral blood compared with patients treated with GD2.CART cells without C7R.

Despite the encouraging advancements of Monje et al., significant challenges remain. The limited sample size of the study and single-institution design constrain the generalizability of its findings, and the exclusion of patients with bulky thalamic or cerebellar disease further narrows its applicability. Moreover, the immunosuppressive tumor microenvironment continues to impede durable responses, as evidenced by elevated TGF-β levels in non-responders. Mechanisms of resistance, including limited persistence and immune suppressive mechanisms warrant further investigation to optimize therapeutic durability.

In late 2024, the FDA designated GD2-CAR-T-cell therapy for DMG as a *Regenerative Medicine Advanced Therapy (RMAT)*, underscoring its potential transformative impact that reflects the agency’s assessment that early clinical evidence demonstrates substantial improvement over existing therapies for DMG and its development should be accelerated. Future directions should refine delivery strategies, such as ICV-only administration, while addressing the logistical complexities of repeated infusions. Combinatorial approaches incorporating immune checkpoint inhibitors or agents targeting the tumor microenvironment may offer synergistic enhancements to CAR-T-cell efficacy. These early results suggest promise for continued targeting of optimized GD2 CAR-Ts in DMG and other cancers as we await more data from multiple ongoing clinical trials (ie, NCT05298995, NCT05544526).

B7-homolog 3 protein (B7-H3), or CD276, has also been found to be upregulated in multiple cancers, including DMG.^93^^,^^107^ An open, single-site, Phase 1 trial (NCT04185038) is evaluating B7-H3 CAR-T cells in pediatric CNS tumors including DMGs. Intracerebroventricular administration was repeated as outpatients with an alternate week dosing regimen for a total of 40 doses for the first 3 patients.^108^ Preliminary results pointed to an increased immune engagement by cytokine evaluation in serum and CSF, CAR-T persistence in the CNS, and ICV tolerability.

In early 2025, the final results of this first-in-human phase 1 trial were published.^109^ Twenty-three pediatric and young adult patients were enrolled, with 21 receiving multiple ICV infusions without prior lymphodepletion. The therapy was well-tolerated overall. Primarily grade 1-2 adverse events were documented, and only one dose-limiting toxicity was observed. Pharmacodynamic analyses demonstrated local CAR-T cell expansion, cytokine induction, and evidence of immune activation in the cerebrospinal fluid. Median OS from diagnosis was 19.8 months, notably longer than the historical median of ∼11 months, with several patients surviving beyond 30-44 months. Given these encouraging results, a multisite phase 2 trial is planned to more rigorously assess clinical efficacy.

In recognition of these promising outcomes, the FDA granted the B7-H3-targeted CAR-T cell product (SCRI-CARB7-H3v1) both *Breakthrough Therapy Designation* in April 2025 and *RMAT Designation* later that month. Collectively, they mark a major regulatory milestone and underscore the therapeutic potential of ICV-delivered CAR-T cells in DMG, as further preclinical efforts continue to refine CAR design, enhance CNS trafficking, and explore combinatorial immunomodulatory approaches.

Although B7-H3 CAR-T therapy has demonstrated remarkable early phase success, therapeutic resistance and incomplete responses remain major challenges, which include tumor heterogeneity and antigen escape. These limitations underscore the need for multi-antigen approaches that can sustain antitumor efficacy across diverse tumor subclones. Thus, this Seattle-based research group has recently initiated a first-in-human Phase I CAR-Trial (BrainChild-04, NCT05768880) for children and young adults with DMG as well as Relapsed/Refractory (R/R) CNS tumors, targeting 4 TAAs: B7-H3, HER2, EGFR806, and IL-13-Rα2. Patients will receive a heterogenous CAR-T population expressing 4 distinct CARs by different subsets to address challenges associated with tumor heterogeneity. This “Quad CAR-T” approach holds tremendous promise to reduce the likelihood of immune evasion and broaden tumor coverage within heterogeneous DMG lesions.

Overall, CAR-T cell therapy for DMG is widely regarded as one of the most encouraging avenues for a cure to DMG. Rapid progress is noticeable as clinical trials are refining approaches from a single bolus IV infusion to repeated, fractionated locoregional infusions of multi-antigen targeting CAR-Ts, which have demonstrable positive tolerability and anti-tumor profiles with effectiveness over antigen escape.^93^ Challenges that lie ahead include the limited accessibility of living tumor samples for TAA phenotyping and treatment stratification and the financially demanding production costs.^93^

### Vaccines

Tumor vaccines are a category of immunotherapy that induces a T-cell response to tumor-specific antigens (TSAs). Vaccine epitopes are typically conjugated to immunostimulatory biological adjuvants, which enhance the potency of the adaptive immune response. A list of the current vaccines in clinical trial for the treatment of DMG are summarized in Table S4.

Tumor vaccine design is multi-faceted and still evolving. It must address challenges such as inter- and intra-tumor heterogeneity, the classification of epitopes that influence the immune response, and key considerations for antigen selection, including their role in cellular survival and immunogenicity. Among the many promising antigen candidates for DMG, the H3K27M mutation is most notable given its inherent specificity. Preclinical studies of an H3.3K27M peptide vaccine demonstrated a robust T-cell-mediated immune response, leading to the initiation of a phase I clinical trial in combination with polyIC ± nivolumab (NCT02960230).^110^ Preliminary evidence shows that the treatment is well tolerated, and 6 out of 18 patients developed an immune response.^111^ Two other H3.3K27M peptide vaccine trials are currently ongoing in combination with IR (NCT04749641) and IR with the anti-PD-L1 antibody atezolizumab (NCT04808245). Results have yet to be posted.

Aside from peptide vaccines, novel vaccination modalities are increasingly receiving attention for high grade gliomas. Professional antigen presenting cells (APCs) are a critical player in tumor-specific vaccination with several current trials seeking to evaluate the efficacy of APCs that have been transfected with tumor antigens (ie, whole tumor cells, peptide extract, or tumor-derived RNA). Since the discovery of H3.3K27M, a multitude of studies have investigated the use of H3.3K27M in APC vaccines.^8^^,^^112-114^ Moreover, RNA lipid particle-based immunotherapies are becoming heavily studied in glioblastoma, with the goal of developing a “universal” vaccine. That is, the vaccine is not meant to selectively target mutated cells of cancer but instead simply prompt a strong immune response.^115^^,^^116^

Despite the clinical benefit of tumor vaccines remaining elusive to date, novel strategies remain an important pillar of immunotherapeutic development for DMG, especially as an adjuvant.

### Oncolytic Viruses

Oncolytic viruses are an emerging class of immunotherapies that promote selective tumor lysis and induction of anti-tumor immunity.^117^ Some OVs that are derived from nature (Seneca Valley virus, Newcastle virus, and reovirus) have immediate anti-tumor effects, while most (oncolytic herpes virus, oncolytic adenovirus, and oncolytic measles virus) have been modified for specificity, immunogenicity, and safety.^118^ Fundamentally, cancer cells are thought to be more sensitized to OV infection and viral replication, secondary to intrinsic abnormalities of cell signaling and anti-viral machinery.^119^ OV-mediated cytolysis is a coordinated series of events that depend on viral entry, viral replication, and the secondary immune response to the virus.^118^^,^^120^ A list of the current OV clinical trials for the treatment of DMG are summarized in Table S5.

The exploitation of specific surface proteins is critical to enhancing OV entry and subsequent oncolysis. Nectin-1 (CD111) is significantly higher in pediatric brain tumor xenografts compared to adult glioblastoma, which prompted increased investigation into oncolytic herpes virus (oHSV) virotherapy.^121^ CD111 is a receptor molecule vital to promoting oHSV entry and is used to predict sensitivity to herpes oncolytic therapy.^122^ HSV-G207 is a genetically engineered HSV-1 mutant designed for cancer treatment.^123^ It originated from HSV mutants such as HSV-1716 and HSV-R3616, which have both copies of the RL1 gene deleted. This gene encodes ICP34.5, a protein critical for the virus’s neurovirulence and immune evasion by counteracting host defenses like PKR-mediated protein synthesis shutdown and autophagy. Without ICP34.5, these mutants are unable to replicate in most normal cells but retain selective replication in tumor cells, where antiviral responses are often impaired, triggering an oncolytic effect. To further enhance safety and tumor specificity, HSV-G207 was created by inserting the *Escherichia coli* lacZ gene into the virus’s ICP6 gene, which encodes ribonucleotide reductase, thereby weakening the virus further while retaining its ability to target and destroy cancer cells. A phase I study with intratumorally delivered G207 as a treatment for pHGG (NCT02457845) reported tolerable safety and promising efficacy results with radiographic, neuropathological, and/or clinical responses in 11 out of 12 patients.^124^ The median OS was extended from 5.6 months in historical controls to 12.2 receiving G207. In addition, there is currently a clinical trial utilizing oHSV G207 in recurrent pediatric cerebellar brain tumors.^125^ Of note, in any clinical trial, no patient, pediatric or adult, treated with G207 has suffered from virus-associated neurotoxicity such as encephalitis.

Oncolytic adenoviruses have been genetically altered to selectively infect tumor cells by targeting oncogenic mutations such as those in the retinoblastoma tumor suppressor (Rb) signaling pathway, which is present in most gliomas.^126^^,^^127^ For example, Fueyo and colleagues engineered a tumor-selective adenovirus by modifying the virus’s early region 1A (E1A) protein, which in wild-type (WT) adenovirus binds to the Rb protein and release E2F-family transcription factors, thereby promoting cell cycle progression and transcriptional activation.^127^ By deleting the Rb binding domain in the viral E1A protein, the virus now selectively infects and kills cancer cells, demonstrating *in vitro* and *in vivo* cytolytic activity. Of note, 59% of pHGG cell lines contain Rb mutations, and thus this strategy has garnered attention for use in DMG.^8^ DNX-2401 is a modified oncolytic adenovirus that contains an integrin-binding RGD-4C motif to enhance glioma tropism. A phase I clinical trial for recurrent malignant adult glioma using DNX-2401 (NCT00805376) found a 3-year survival of 20%.^128^ In addition to radiographic signs of inflammation, histopathologic examination of immune markers in post-treatment specimens showed intratumoral T cell infiltration and TIM3 downregulation after treatment. These promising results, in addition to preclinical data specific to DMG, have motivated a phase I/II clinical trial (NCT03178032).^129^^,^^130^ Intratumoral infusion of DNX-2401, followed by radiotherapy in pediatric patients with DMG, led to tumor reduction in 9 out of 12 patients as well as increased infiltrative CD8+/CD4+ T-cells and absent regulatory T cells in the one patient with available tissue from repeat biopsy/autopsy. Impressively, the median OS was 17.8 months compared to the 8-12 months of historical DIPG patients treated with radiation therapy alone.^4^^,^^131^ Nevertheless, the small sample size (*n* = 12) and the lack of a randomized design preclude any conclusion regarding survival, but do provide a rationale for a larger trial even in light of some serious adverse events (*n* = 3).

The efficacy of OV-mediated immunotherapy for brain tumors are heavily dependent on selective delivery. Mesenchymal stem cells (MSCs) can cross the blood-brain barrier (BBB) and localize to areas of brain tumor even with systemic administration.^132^ This homing capacity makes them excellent vectors of anticancer therapeutics. Promising preclinical data from GBM models showed that MSCs carrying a virus delayed its elimination by the host immune system and provided neuroprotection to normal peritumoral tissue^133^ and thus, led to a phase I clinical trial investigating the safety of OV-carrying MSCs for GBM (NCT03072134). Since then, encouraging preclinical findings have also emerged using DMG patient-derived xenografts (PDX) and intranasal delivery.^134^ Carceller and colleagues administered Celyvir, an autologous mesenchymal stem cell infected with ICOVIR-5 (an oncolytic adenovirus that selectively replicates in cancer cells) by means of superselective intra-arterial delivery, in a 9 year old patient diagnosed with DMG.^135^ They reported impressive tolerance to the 2 administrations with no acute or delayed adverse effect, which underscores the feasibility of this technique for the delivery of virotherapies and/or cellular therapies in midline regions. This experimental approach is particularly inspiring for the development of less invasive methods of CNS delivery.

### Controlling Toxicities

A vital aspect of achieving success with immunotherapies in pHGGs is inducing a localized inflammatory response.^136^ That said, inflammatory responses and toxicities from acute therapy can be harmful and potentially life-threatening especially in compact, eloquent areas such as the brainstem where immune-related edema is not well-tolerated. Toxicities are an important consideration with all immunotherapy, but have rarely been reported for DMG tumors.^93^ Cytokine release syndrome is a potentially lethal toxicity from immunotherapies such as CAR-T treatments, and describes a systemic inflammatory episode resulting in fever, rigors, and even organ failure.^93^^,^^137^ It is typically myeloid-mediated through the production of pro-inflammatory cytokines or nitric oxide.^137^ Of note, almost 90% of patients administered IV CAR-T therapy experience some degree of CRS, and nearly 30%-50% of patients may develop more severe symptoms.^93^^,^^137^ Immune effector cell-associated neurotoxicity syndrome (ICANS) is another toxicity documented in several immunotherapy approaches, and is characterized by an array of neurological symptoms such as headache, tremors, and aphasia as well as increased intracranial pressure, hemorrhage, and coma.^93^^,^^137^^,^^138^ Notably, ICANS has not been reported frequently in DMG CAR-T cell trials.

Lastly, TIAN is a newly termed syndrome experienced by patients with CNS malignancy, primarily after immunotherapy treatment. Importantly, TIAN is relevant not only to cellular therapies but to all therapies that seek to stimulate the immune system of the CNS.^139^ As opposed to the generalized and diffuse cerebral edema of severe ICANS, TIAN involves localized inflammation at the tumor site but extends beyond the concept of pseudoprogression, and can lead to significant edema that poses risks such as neuronal dysfunction, increased intracranial pressure (ICP), tissue herniation, and obstruction of CSF flow.^93^^,^^139^ While Type 1 TIAN is used to describe neurological symptoms secondary to mechanical factors, Type 2 is due to primary local neural dysfunction and can even occur in the absence of edema.

Given the increasing application of immunotherapies in DMG, the establishment of distinct subtypes as well as a standardized clinical grading scale for TIAN has become crucial. Unlike CRS and ICANS, the TIAN grading framework accounts for localized, on-target, and anatomy-dependent manifestations such as peritumoral edema, elevated intracranial pressure, and region-specific neural dysfunction. This targeted classification provides a more practical and clinically relevant approach for evaluating CNS-directed immunotherapy toxicities, which facilitates early recognition, guides intervention, and enables consistent comparison across studies.

### Improving Delivery

Despite significant progress in preclinical research and clinical trials on targeted, immunotherapeutic agents, biological barriers within the brain and its tumor microenvironment remain a critical challenge to overcome for improving patient outcomes. In neuro-oncology, the BBB is often termed as the blood-brain tumor barrier (BBTB). Typically, brain tumors are described to disrupt the anatomy as well as function of the BBB and provide openings for drug delivery. Several studies to date have characterized the BBTB of DMG to have a relatively intact BBB that renders systemic drug delivery more challenging than other brain cancers.^140-142^ Thus, it is critical that novel delivery technologies are explored and optimized to bypass or disrupt the DMG BBTB. Over the past 5 years, these have primarily included Ommaya reservoirs, convection enhanced delivery (CED), and focused ultrasound (FUS).^143^

Ommaya reservoirs are dome-shaped silicone reservoirs that are surgically implanted under the scalp, and connects to an indwelling catheter positioned in the ventricles of the brain. While Ommaya reservoirs have been a valuable neurosurgical device for decades, it has recently emerged as a method to administer immunotherapy agents directly into the ventricular CSF spaces for DMG patients. Promisingly, its ability to bypass the BBTB allows for higher drug concentrations to reach the target site, and has been found to decrease systemic toxicities in addition to improving anti-tumor responses (NCT04185038, NCT04196413, NCT04099797, and NCT04196413).^104^^,^^143^ Similar to an Ommaya reservoir, CED is a neurosurgical technique where one or more small catheters are stereotactically placed within the ventricle/CSF spaces or near a tumor to directly administer agents including immunotherapies.^144^^,^^145^ While also invasive, CED offers significant advantages over systemic drug delivery and diffusion, where therapeutics passively permeate tissues, CED utilizes convection to create a local hydrostatic pressure gradient that enables more uniform and widespread drug distribution. Though CED has been routinely proven safe for most agents, its efficacy has been impeded by the multitude of parameters that require optimization, including infusion flow rate, distribution area, and number of infusions.^146^ For example, a phase I trial with CED of IL13-Pseudomonas toxin in children with DIPG/DMG (NCT00088061) found the treatment to be safe with no radiographic evidence of acute or long-term treatment toxicity, but only 2 out of 5 patients had a transient arrest of disease progression.^147^ Focused ultrasound is a minimally invasive drug delivery technique that uses intravenously administered microbubbles and targeted ultrasound energy to transiently disrupt the BBB. The oscillation of circulating microbubbles under ultrasound induces mechanical stress on the vascular endothelium, leading to temporary tight junction opening, decreased efflux transporter activity, and increased vesicular transcytosis. This results in a localized and reversible increase in BBB permeability, with an increase in convective transport within the tumor interstitium. Although not tested yet for immunotherapies in clinical trials of DMG, FUS has shown promise for delivering large molecules such as antibodies and viruses as well as immunomodulatory compounds/proteins.^148-151^ For a more detailed discussion on any of these delivery modalities, refer to Arms et al.^143^

## Future Directions

**Figure 5.**
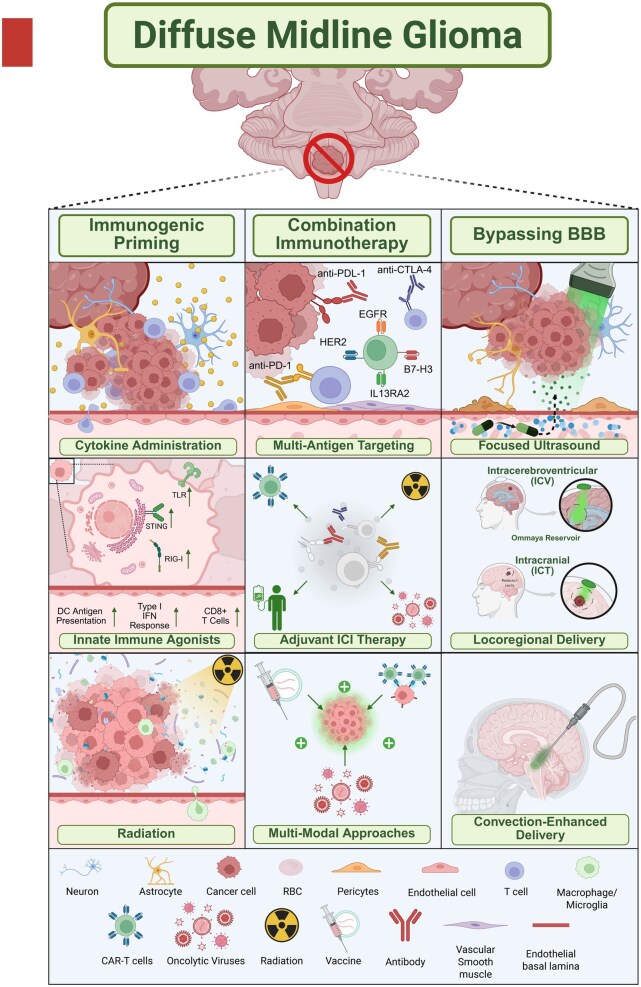
Overcoming the DMG TME. A summary of current approaches to remodel the tumor microenvironment and improve immune response in DMG, including immunogenic priming, multi-antigen and combination immunotherapies, and techniques to bypass the blood-brain barrier for targeted delivery. Created in BioRender. Shah, A. (2025) https://BioRender.com/awne5ez

Diffuse midline glioma carries one of the most devastating prognoses among CNS diseases, and although immunotherapy remains the most promising modality for achieving durable responses, numerous obstacles complicate progress toward improved outcomes (Figure 4). We propose a 3-tiered strategy to overcome the DMG TME in order to sensitize the tumor to immunotherapy (Figure 5). This framework includes immunogenic priming, bypassing the BBB, and combining therapeutic modalities. Immunogenic priming of the DMG TME may involve a range of approaches, including standard of care IR and TLR agonists. Blood-brain barrier bypass can be achieved through neurosurgical interventions such as an Ommaya reservoir or CED, or noninvasively through FUS. Although combining immunotherapies is conceptually appealing, results to date have been complex.^152^ Unreliable TME biomarkers and pronounced tumor heterogeneity further limit the development of well-informed combinations. Despite the large number of new combinatorial trials initiated for DMG each year, serial sampling approaches that generate hypothesis driven biomarkers will facilitate more rational combinations with respect to modality, timing, and scheduling.

Another critical obstacle that DMG research continues to face before clinical translation is the development of robust preclinical models.^153^ Although patient derived cell line models have been instrumental in elucidating key molecular attributes of H3K27M+ DMG cells, their major limitation lies in the inability to accurately study interactions with other cellular components of the brain tumor microenvironment. Moreover, xenografts derived from these models are typically immunodeficient, require orthotopic engraftment (which may artificially disrupt the BBB), and do not faithfully recapitulate the human TME. Consequently, the field has increasingly adopted genetically engineered mouse models (GEMMs), either traditional systems with modification of oncogenes or in utero electroporation (IUE), that provide opportunities to study immune based therapies. Additional efforts include the establishment of DMG organoid models and co-culture systems that more reliably reproduce the TME, as well as the development of DMG models in larger species.

Recent studies that have characterized these immunocompetent GEMMs, together with analyses of patient samples, indicate that the DMG TME is less uniform than previously appreciated and that some subtypes may not be immunologically cold. Importantly, emerging work is defining how different oncohistones (H3.1 or H3.3 K27M) influence the TME in both models and patient tissue. A deeper understanding of how the mutational profile of DMG shapes its TME is essential. For example, specific mutations may correlate with tumor inflammation signatures and could be leveraged as biomarkers to improve responses to ICI treatment.

With encouraging data from trials and accompanying accelerated approval designations from the FDA, optimism surrounding immunotherapies for DMG is at an all-time high. As additional later phase clinical trial data emerge, they will likely inform refinements in standard of care and help define synergistic combinations among immunotherapy modalities.

## Supplementary Material

noag014_Supplementary_Data
