# Supplementary material for: Deciphering the tumor microenvironment and role of immunotherapy in diffuse midline glioma: A scoping review
Source: Neuro Oncol. 2026 Feb 2;28(4):829–46. doi: 10.1093/neuonc/noag014 (PMC13128486; doi:10.1093/neuonc/noag014)
Supplement: noag014_Supplementary_Data [file noag014_supplementary_data.zip › Supplement 3.docx]

DIPG/DMG INTEGRATATION WITH NEURAL NETWORKS

Cancer neuroscience is the study of the complex interactions between the nervous system and cancer^1^. This interdisciplinary approach incorporates developmental biology, oncology, and immunology to ultimately elucidate more efficacious treatment paradigms. Teams led by Stanford University and the University of Heidelberg have sought to explore the functional and mechanistic parallels between neuroglial communication during development and within the tumor microenvironment. They uncovered novel mechanisms of neuron-to-glioma communication via paracrine signaling and synaptic interaction, and have examined intrinsic glioma-to-glioma communications to include the remodeling of its electrochemical landscape to promote its own growth^2^. Of note, they proposed a triangular crosstalk that occurs between glioma cells, neurons, and immune cells as a framework to understand how these cells cooperate to undermine current therapeutic interventions. At the forefront of this work, teams are applying this novel strategy to improve the efficacy of immunotherapies against DMG. This section will examine the current literature in cancer neuroscience to explain the various ways DMG hijacks communication mechanisms of CNS development and how this triangular framework may be being leveraged immunologically against DMG.

Neuronal Paracrine Signaling Promotes Glioma Growth

As mentioned previously, the cell of origin of DMG/DIPG is predominantly oligodendrocyte precursor cells (OPCs)^3, 4^. During development, OPC proliferation and maturation have been shown to be regulated by the activity of neuronal circuits^5^. It is on this backdrop that in 2015 Venkatesh and colleagues first identified neuroligin-3 (NLGN3) as a paracrine factor that promotes HGG proliferation and growth *in vivo*.^6^ Utilizing novel model systems for neuronal activation under optogenetic-control including patient-derived HGG xenograft models and cortical slices, the authors found a striking influence of neuronal activity on tumor burden during the exponential growth phase. After a series of biochemical assays, Venkatesh and colleagues narrowed down the effectors of this neuron-mediated mitogenic response to be secreted proteins. Proteomic analyses revealed the top hits to include brain-derived neurotrophic factor (BDNF), the known glioma mitogen 78 kDa glucose-regulated protein (GRP78), and, most potently, NLGN3. Mechanistically, they uncovered that secreted neuroligin-3 promotes a feedforward expression of NLGN3 within the glioma cell itself through recruitment of the PI3K-mTOR pathway. These neuronal activity-regulated paracrine signaling mechanisms influence a wide range of glioma types, including DIPG/DMG^6^, IDH- WT GBM^6^, IDH-mutant oligodendroglioma^6^, and NF1-mutant optic pathway glioma.^7^ Validating its clinical significance in human glioma pathophysiology, the authors found NLGN3 mRNA levels to be inversely correlated with GBM patient overall survival.

In 2017, further work sought to elucidate outstanding questions such as the necessity of NLGN3 for glioma growth, the proteolytic mechanism of NLGN3 secretion, and the further molecular consequences of NLGN3 secretion in glioma cells.^8^ Utilizing *Nlgn3* knockout xenograft mouse models, they robustly demonstrated that microenvironmental NLGN3 is necessary for HGG growth in numerous PDGCs. Leveraging phosphoproteomics and RNA sequencing, NLGN3 exposure was shown to cause focal adhesion kinase (FAK) phosphorylation and numerous phosphorylation events classically downstream of FAK as well as upregulation of oncogenes including *PDGFA*, *TTYH1,* and several potassium channel genes. With clinical translation in mind, the authors next sought to determine the enzyme responsible for NLGN3 secretion, and if inhibition was possible. After identifying the ADAM10 sheddase as potential target, DIPG xenografts were treated with the ADAM10 inhibitor and exhibited pronounced reduction in tumor size. ^8^

Gliomas as an Integrated Part of the Neuronal Circuit

In addition to oncogenic modulation, NLGN3 exposure was also noted to induce glioma expression of numerous synaptic genes.^8^ Thus, in 2019, Venkatesh and colleagues sought to explore this phenomenon further: do gliomas engage in synaptic communication?^9^ Starting with transcriptomic datasets, they observed broad expression of glutamate receptor genes and postsynaptic structural genes in malignant glioma cells. As demonstrated by electron and immuno-electron microscopy, primary tumor cells from glioblastoma samples were found to form structural, NLGN3-dependent synapses with neighboring neurons. Further, in pediatric glioma xenograft models including DIPG, glioma cells were shown to receive functional excitatory synaptic input from neurons, confirmed by patch-clampelectrophysiological recordings showing neuronal action potential–dependent currents. Recordings showed that glioma cells receive excitatory signals from neurons through calcium-permeable AMPA (α-amino-3-hydroxy-5-methyl-4-isoxazole propionic acid) receptors, and both miniature currents and calcium imaging confirmed these as real, functional synapses. Some glioma cells exhibited prolonged (>1 s), non-synaptic currents resembling those seen in astrocytes, which were unaffected by AMPA receptor blockade. However, they were triggered by elevated extracellular potassium and reduced by potassium channel inhibition, indicating that this second type of glioma currents are primarily driven by potassium flux during neuronal activity. These potassium-evoked currents are amplified in cells connected by gap junction-coupled networks, allowing potassium-driven depolarization to spread, with about 40% of DMG cells showing this response and 5–10% instead exhibiting synaptic excitatory postsynaptic currents (EPSCs).

In pediatric glioma xenograft models, they showed neuron-to-glioma synaptic activity through AMPA receptors was a major driver of tumor growth, and blocking these currents or gap junction–mediated potassium signaling significantly reduced tumor proliferation^6^. The researchers used intraoperative electrocorticography in awake GBM patients to record high gamma (70–110 Hz) activity, a marker of neuronal firing, and found markedly elevated signals in tumor-infiltrated cortex compared to healthy-appearing brain. They observed similar neuronal hyperexcitability in pediatric glioma xenografts, supporting the idea that hyperactive neurons in the tumor microenvironment can drive glioma progression by enhancing both potassium-mediated prolonged currents and synaptic AMPA receptor–mediated inputs to glioma cells.

In parallel, a second group, Venkataramani and colleagues, corroborated these core principles in adult glioblastoma when they similarly demonstrated functional glutamatergic synapses between neurons and glioma cells.^10^ Using patient-derived xenografts, they identified both AMPA receptor–mediated synaptic currents and gap junction–mediated network activity, closely paralleling the dual mechanisms observed in the pediatric glioma models of Venkatesh et al. This reinforced the idea that disrupting neuron-to-glioma electrochemical communication could be an effective therapeutic strategy across glioma subtypes.

Activity-Dependent Plasticity in Pediatric Glioma

As the role of the nervous system in the regulation of cancer became increasingly appreciated, more complex questions came to the forefront. Neuronal activity had already been found to drive tumor progression through paracrine signaling factors (NLGN3, BDNF) and electrophysiologically functional neuron-to-glioma synapses (AMPA receptors). Next questions focused on the roles of adaptive plasticity and epigenetic dysregulation in neuron-to-glioma synapse formation. Importantly, in the healthy brain, activity-regulated secretion of BDNF is known to promote adaptive plasticity of synaptic connectivity and strength.

In 2023, Taylor *et al.* investigated whether malignant synapses undergo adaptive changes in response to neuronal activity, and identified brain-derived neurotrophic factor (BDNF)–TrkB signaling as a central driver of this process.^2^ Although pediatric gliomas like DIPG/DMG lack BDNF expression, they display high levels of the BDNF receptor TrkB (NTRK2), enabling them to respond to activity-dependent BDNF release from surrounding neurons^2^.

Through optogenetic stimulation and *in vivo* xenograft models, the authors showed that neuronal activity enhances glioma synaptic strength and consequent glioma proliferation via TrkB^2^. Genetic deletion or pharmacological inhibition of TrkB reduced tumor growth, diminished neuron–glioma synapse number, weakened synaptic currents, and prolonged survival. Mechanistically, BDNF–TrkB signaling increased AMPA receptor subunit trafficking (GluA2/3/4) to the glioma cell membrane via CAMKII, which strengthened excitatory synaptic currents^2^. They went on to show that larger amplitude depolarization of the glioma membrane – resulting from stronger synaptic currents - is itself growth-promoting in glioma cells through voltage-sensitive mechanisms that remain to be fully elucidated^2^. This increase in synaptic strength occurred alongside an increase in the number of neuron–to-glioma synapses, amplifying excitatory drive to the tumor^2^.

These results reveal that gliomas not only passively receive neuronal input but actively reinforce and expand malignant synapses through activity-regulated plasticity mechanisms. Analogous to learning and memory processes in healthy brain circuits, gliomas hijack the BDNF–TrkB–AMPAR axis to sustain excitatory signaling that fuels their proliferation^2^. The work highlights TrkB inhibition as a promising therapeutic strategy, particularly as clinically approved TRK inhibitors could be repurposed for non–NTRK fusion gliomas.

Given the known epigenetic dysregulation that drives DMG, Zhang and colleagues sought to understand if and how this affected neuron-glioma interactions. In 2024, they reported that dysregulated epigenetic mechanisms increase neuron-to-glioma synapses in DMG, specifically the CDH2-FOSL1 axis is a key driver of synaptic and axon guidance gene expression in the H3.1K27M subtype.^11^ Through *in vitro* co-cultures, xenograft models, and optogenetic studies, the authors showed CDH2 binds directly at synaptic and axon guidance gene loci and colocalizes with FOSL1 to alter histone modifications and regulate neuron-glioma interactions. Genetic depletion of CDH2 and FOSL1 reduced H3K27ac and H3K4me3 and increased H3K27me3.^11^. This inhibited synaptic protein expression, axon guidance gene expression, and neuron-induced tumor proliferation.^11^. Interestingly, CHD2 silencing did not affect expression of known regulators of neuron-glioma interactions *NLGN3*, *BDNF*, *TRKB*.^11^. Despite this epigenetic axis being highly targetable, different subtypes likely exhibit distinct epigenetic mechanisms to mediate these interactions.

In summary, these works suggest that DMG not only co-opts existing mechanisms of adaptive plasticity but also existing epigenetic modulatory mechanisms to further reinforce the feedback mechanisms that alter neuroglial interactions at the cellular level. This underscores yet another way DMG leverages the neurophysiology of the tumor microenvironment in its favor. Whether there are other targetable mechanisms of adaptive plasticity or epigenetic regulation in DMG remains unclear.

Glioma subtype-specific GABA biology and Importance of Neuromodulatory Circuits in DIPG/DMG

Although Labrakakis *et al.* first hinted in the late 1990s that functional GABA_A_ receptors could play a role in glioma growth,^12^ the role of non-glutamatergic neurotransmission in neuron–to-glioma synapses was still largely a blind spot. That changed in 2025, when two landmark papers unveiled both a far more complete and complex picture where gliomas manipulate additional neurotransmitter classes of neurons to their advantage.

Using whole-cell patch-clamp recordings, *in vivo* optogenetics, and xenografts, Barron and colleagues identified functional GABAergic neuron–to–glioma synapses in DMGs mediated by GABA_A_ receptors.^13^ Similar to what occurs in normal neural precursor cells and healthy OPCS, in DMG cells NKCC1 transporter activity elevates intracellular chloride, making GABAergic input depolarizing (excitatory) rather than hyperpolarizing (inhibitory), thereby promoting proliferation. Pharmacologically enhancing GABA signaling with lorazepam further increased tumor growth and reduced survival in DMG models. In contrast, IDH-WT hemispheric high-grade gliomas exhibited minimal depolarizing GABAergic currents, and lorazepam had no effect on their growth. Thus, this indicates a subtype-specific, growth-promoting role for GABAergic synaptic communication in H3K27M-altered DMGs, with great relevance to clinical practice. Lorazepam is commonly used in children with DMG for nausea, anxiety, claustrophobia during MRI scans, and other indications. While it will remain an important medication for palliative care, especially in low doses, potential risks of high-dose lorazepam in H3K27-altered DMG patient populations should be further evaluated in clinical studies.

Beyond classical excitatory and inhibitory synapses, recent work by Drexler and colleagues has also identified neuromodulatory brainstem circuits as potent regulators of DMG progression.^14^ While glutamatergic signaling is known to drive proliferation of both oligodendrocyte precursor cells (OPCs) and gliomas, the role of long-range neuromodulatory inputs to the midline brain regions where DMGs arise had been understudied.

Using *in vivo* optogenetic stimulation in xenograft models, the Drexler and colleagues showed that midbrain cholinergic neurons in the pedunculopontine nucleus (PPN) selectively promote glioma growth in the pons, whereas cholinergic neurons in the laterodorsal tegmentum nucleus (LDT) stimulate proliferation in the thalamus. These effects were anatomically and circuit-specific, reflecting the natural projection patterns of each nucleus. Importantly, DMG-bearing mice exhibited a progressive increase in acetylcholine release and cholinergic neuron firing rates over the course of disease, suggesting a feedback loop between tumor progression and cholinergic activity.

Mechanistic dissection revealed that acetylcholine acts directly on DMG cells to drive proliferation. In co-culture assays, cholinergic neurons enhanced DMG growth, with acetylcholine treatment alone replicating this effect. Single-cell RNA sequencing of human DMG samples revealed high expression of CHRM1 and CHRM3, encoding M1 and M3 muscarinic acetylcholine receptors, in OPC-like glioma cell populations. Genetic knockout or pharmacological inhibition (VU0255035, 4-DAMP) in xenografts completely abolished the proliferative response to cholinergic stimulation, confirming a receptor-specific mechanism.

Together, these findings expand the framework of glioma neurobiology beyond glutamatergic and GABAergic synaptic transmission to include neuromodulatory neurotransmitter systems with region-specific control over tumor growth.

Crosstalk Between Neurons, Gliomas, and the Immune System

As discussed above, CNS is an immunologically active tissue. A triangular dynamic has been described in cancer neuroscience involving neurons, immune cells, and glioma cells that modulates the tumor immune microenvironment, pro- and anti- tumor immunity, and susceptibility to immunotherapy.^15, 16^ However, many aspects remain unexplored.

An early example of neuro-immune integration has been demonstrated in experiments with Nf1 optic low-grade glioma mice where paracrine factor secretion acted on T cells to trigger microglial secretion of the CCL5.^17^ More recently, Nejo et al. delineated a mechanistic link between heightened neuron–glioma connectivity and regional immunosuppression in glioblastoma, with connected tumor niches enriched for anti-inflammatory TAMs.^18^ In preclinical models, loss of tumor-derived thrombospondin-1 (TSP1/Thbs1) or pharmacologic dampening of glutamatergic signaling reduces synaptogenesis and neuronal hyperexcitability, which in turn restored antigen-presentation programs, shifted TAMs toward pro-inflammatory states with increased CD8⁺ T-cell infiltration, and prolonged survival. Even as these studies highlight the neuron–glioma–immune axis as a tractable target for immunotherapy, neuro–immune integration in pediatric HGGs remains poorly characterized and represents an important direction for future work.

In summary, DMG integration into synaptic networks is pervasive and robust. Increased expression of synaptic proteins and enhanced neuronal excitability cooperate to drive glioma proliferation in a durable positive feedback loop. In addition to glioma integration into neural circuits, gliomas mimic the proliferative mechanisms of developing glia to modify glioma-to-glioma interactions in its favor. These findings provide a conceptual framework through which novel combinatorial strategies can be elucidated. Although many strides have been made in understanding the various connections of this triangular network, further investigation is warranted into uncovering possible therapeutic synergies between immune cells, neurons, and glioma cells that leverage the electrochemical microenvironment against DMG.

References:

1. Mancusi R, Monje M. The neuroscience of cancer. Nature. 2023;618(7965):467-79. doi: 10.1038/s41586-023-05968-y.

2. Taylor KR, Barron T, Hui A, Spitzer A, Yalçin B, Ivec AE, Geraghty AC, Hartmann GG, Arzt M, Gillespie SM, Kim YS, Maleki Jahan S, Zhang H, Shamardani K, Su M, Ni L, Du PP, Woo PJ, Silva-Torres A, Venkatesh HS, Mancusi R, Ponnuswami A, Mulinyawe S, Keough MB, Chau I, Aziz-Bose R, Tirosh I, Suvà ML, Monje M. Glioma synapses recruit mechanisms of adaptive plasticity. Nature. 2023;623(7986):366-74. doi: 10.1038/s41586-023-06678-1.

3. Monje M, Mitra SS, Freret ME, Raveh TB, Kim J, Masek M, Attema JL, Li G, Haddix T, Edwards MS, Fisher PG, Weissman IL, Rowitch DH, Vogel H, Wong AJ, Beachy PA. Hedgehog-responsive candidate cell of origin for diffuse intrinsic pontine glioma. Proc Natl Acad Sci U S A. 2011;108(11):4453-8. Epub 20110301. doi: 10.1073/pnas.1101657108. PubMed PMID: 21368213; PMCID: PMC3060250.

4. Taylor KR, Monje M. Neuron-oligodendroglial interactions in health and malignant disease. Nat Rev Neurosci. 2023;24(12):733-46. Epub 20231019. doi: 10.1038/s41583-023-00744-3. PubMed PMID: 37857838; PMCID: PMC10859969.

5. Gibson EM, Purger D, Mount CW, Goldstein AK, Lin GL, Wood LS, Inema I, Miller SE, Bieri G, Zuchero JB, Barres BA, Woo PJ, Vogel H, Monje M. Neuronal Activity Promotes Oligodendrogenesis and Adaptive Myelination in the Mammalian Brain. Science. 2014;344(6183):1252304. doi: doi:10.1126/science.1252304.

6. Venkatesh HS, Johung TB, Caretti V, Noll A, Tang Y, Nagaraja S, Gibson EM, Mount CW, Polepalli J, Mitra SS, Woo PJ, Malenka RC, Vogel H, Bredel M, Mallick P, Monje M. Neuronal Activity Promotes Glioma Growth through Neuroligin-3 Secretion. Cell. 2015;161(4):803-16. Epub 20150423. doi: 10.1016/j.cell.2015.04.012. PubMed PMID: 25913192; PMCID: PMC4447122.

7. Pan Y, Hysinger JD, Barron T, Schindler NF, Cobb O, Guo X, Yalçın B, Anastasaki C, Mulinyawe SB, Ponnuswami A, Scheaffer S, Ma Y, Chang K-C, Xia X, Toonen JA, Lennon JJ, Gibson EM, Huguenard JR, Liau LM, Goldberg JL, Monje M, Gutmann DH. NF1 mutation drives neuronal activity-dependent initiation of optic glioma. Nature. 2021;594(7862):277-82. doi: 10.1038/s41586-021-03580-6.

8. Venkatesh HS, Tam LT, Woo PJ, Lennon J, Nagaraja S, Gillespie SM, Ni J, Duveau DY, Morris PJ, Zhao JJ, Thomas CJ, Monje M. Targeting neuronal activity-regulated neuroligin-3 dependency in high-grade glioma. Nature. 2017;549(7673):533-7. Epub 20170920. doi: 10.1038/nature24014. PubMed PMID: 28959975; PMCID: PMC5891832.

9. Venkatesh HS, Morishita W, Geraghty AC, Silverbush D, Gillespie SM, Arzt M, Tam LT, Espenel C, Ponnuswami A, Ni L, Woo PJ, Taylor KR, Agarwal A, Regev A, Brang D, Vogel H, Hervey-Jumper S, Bergles DE, Suvà ML, Malenka RC, Monje M. Electrical and synaptic integration of glioma into neural circuits. Nature. 2019;573(7775):539-45. doi: 10.1038/s41586-019-1563-y.

10. Venkataramani V, Tanev DI, Strahle C, Studier-Fischer A, Fankhauser L, Kessler T, Körber C, Kardorff M, Ratliff M, Xie R, Horstmann H, Messer M, Paik SP, Knabbe J, Sahm F, Kurz FT, Acikgöz AA, Herrmannsdörfer F, Agarwal A, Bergles DE, Chalmers A, Miletic H, Turcan S, Mawrin C, Hänggi D, Liu H-K, Wick W, Winkler F, Kuner T. Glutamatergic synaptic input to glioma cells drives brain tumour progression. Nature. 2019;573(7775):532-8. doi: 10.1038/s41586-019-1564-x.

11. Zhang X, Duan S, Apostolou PE, Wu X, Watanabe J, Gallitto M, Barron T, Taylor KR, Woo PJ, Hua X, Zhou H, Wei H-J, McQuillan N, Kang K-D, Friedman GK, Canoll PD, Chang K, Wu C-C, Hashizume R, Vakoc CR, Monje M, McKhann GM, II, Gogos JA, Zhang Z. CHD2 Regulates Neuron–Glioma Interactions in Pediatric Glioma. Cancer Discovery. 2024;14(9):1732-54. doi: 10.1158/2159-8290.Cd-23-0012.

12. Labrakakis C, Patt S, Hartmann J, Kettenmann H. Functional GABA(A) receptors on human glioma cells. Eur J Neurosci. 1998;10(1):231-8. doi: 10.1046/j.1460-9568.1998.00036.x. PubMed PMID: 9753131.

13. Barron T, Yalçın B, Su M, Byun YG, Gavish A, Shamardani K, Xu H, Ni L, Soni N, Mehta V, Maleki Jahan S, Kim YS, Taylor KR, Keough MB, Quezada MA, Geraghty AC, Mancusi R, Vo LT, Castañeda EH, Woo PJ, Petritsch CK, Vogel H, Kaila K, Monje M. GABAergic neuron-to-glioma synapses in diffuse midline gliomas. Nature. 2025;639(8056):1060-8. doi: 10.1038/s41586-024-08579-3.

14. Drexler R, Drinnenberg A, Gavish A, Yalçin B, Shamardani K, Rogers AE, Mancusi R, Trivedi V, Taylor KR, Kim YS, Woo PJ, Soni N, Su M, Ravel A, Tatlock E, Midler A, Wu SH, Ramakrishnan C, Chen R, Ayala-Sarmiento AE, Fernandez Pacheco DR, Siverts LA, Daigle TL, Tasic B, Zeng H, Breunig JJ, Deisseroth K, Monje M. Cholinergic neuronal activity promotes diffuse midline glioma growth through muscarinic signaling. Cell. 2025. doi: 10.1016/j.cell.2025.05.031.

15. Winkler F, Venkatesh HS, Amit M, Batchelor T, Demir IE, Deneen B, Gutmann DH, Hervey-Jumper S, Kuner T, Mabbott D, Platten M, Rolls A, Sloan EK, Wang TC, Wick W, Venkataramani V, Monje M. Cancer neuroscience: State of the field, emerging directions. Cell. 2023;186(8):1689-707. doi: 10.1016/j.cell.2023.02.002. PubMed PMID: 37059069; PMCID: PMC10107403.

16. Song K-W, Lim M, Monje M. Complex neural-immune interactions shape glioma immunotherapy. Immunity. 2025;58(5):1140-60. doi: 10.1016/j.immuni.2025.04.017.

17. Solga AC, Pong WW, Kim KY, Cimino PJ, Toonen JA, Walker J, Wylie T, Magrini V, Griffith M, Griffith OL, Ly A, Ellisman MH, Mardis ER, Gutmann DH. RNA Sequencing of Tumor-Associated Microglia Reveals Ccl5 as a Stromal Chemokine Critical for Neurofibromatosis-1 Glioma Growth. Neoplasia. 2015;17(10):776-88. doi: 10.1016/j.neo.2015.10.002. PubMed PMID: 26585233; PMCID: PMC4656811.

18. Nejo T, Krishna S, Yamamichi A, Lakshmanachetty S, Jimenez C, Lee KY, Baker DL, Young JS, Chen T, Phyu SSS, Phung L, Gallus M, Maldonado GC, Okada K, Ogino H, Watchmaker PB, Diebold D, Choudhury A, Daniel AGS, Cadwell CR, Raleigh DR, Hervey-Jumper SL, Okada H. Glioma-neuronal circuit remodeling induces regional immunosuppression. Nature Communications. 2025;16(1):4770. doi: 10.1038/s41467-025-60074-z.
