# Supplementary material for: Deciphering the tumor microenvironment and role of immunotherapy in diffuse midline glioma: A scoping review
Source: Neuro Oncol. 2026 Feb 2;28(4):829–46. doi: 10.1093/neuonc/noag014 (PMC13128486; doi:10.1093/neuonc/noag014)
Supplement: noag014_Supplementary_Data [file noag014_supplementary_data.zip › DMG Tables 4.0.docx]

**Table Legends (for Online Supplement):**

Supplementary Table 1. Immune landscape by DMG molecular subtype.

Known immunological characteristics of DMG molecular subtypes.

Supplementary Table 2. Recent Immune Checkpoint Blockade Trials in DIPG/DMG

* denotes a combinatorial regimen.

Supplementary Table 3. Recent Adoptive Cell Transfer Trials in DIPG/DMG

* denotes a combinatorial regimen.

Supplementary Table 4. Recent Vaccine Trials in DIPG/DMG

* denotes a combinatorial regimen.

Supplementary Table 5. Recent Oncolytic Virus Trials in DIPG/DMG

* denotes a combinatorial regimen.

| Molecular Subtype | Differences in Immune Landscape |
| --- | --- |
| H3 WT | - Non-Mutant - Associated with a more inflammatory expression profile compared to H3 altered tumors |
| H3.3K27M | - Concomitant TP53/PGFR alteration^154^ - Limited capability of macrophage polarization^44^ - Most disease-associated microglia of any DMG subtype^64^ - Considered “coldest” immune phenotype^45^ - Associated with more immunosuppressive TME than H3.1 tumors |
| H3.1K27M | - Concomitant ACRV1 alteration^155^ - Greater myeloid cell accumulation than H3.3 tumors^44^ - Capable of modulating macrophage polarization^44^ - Associated with a more immunologically responsive TME |

Supplementary Table 1. Immune landscape by DMG molecular subtype.

Known immunological characteristics of DMG molecular subtypes.

| NCT Number | Study Title | Phases | Study Status | Interventions | Start Date | End Date |
| --- | --- | --- | --- | --- | --- | --- |
| NCT04943848 | rHSC-DIPGVax Plus Checkpoint Blockade for the Treatment of Newly Diagnosed DIPG and DMG | I | Recruiting | Balstilimab and Zalifrelimab | 01/2022 | 12/2025 |
| NCT02750891 | A Study of DSP-7888 in Pediatric Patients With Relapsed or Refractory High Grade Gliomas | I/II | Completed | Novel Drug: DSP-7888 | 04/2016 | 01/2020 |
| NCT01952769 | Anti PD1 Antibody in Diffuse Intrinsic Pontine Glioma | I/II | Unknown | MDV9300 | 02/2014 | 04/2019 |
| NCT05063357 | 131I-omburtamab Delivered by Convection-Enhanced Delivery in Patients With Diffuse Intrinsic Pontine Glioma | I | Withdrawn | 131-I radiolabeled Omburtamab | 03/2022 | 12/2026 |
| NCT03389802 | Phase I Study of APX005M in Pediatric Central Nervous System Tumors | I | Active - Not Recruiting | APX005M - humanized IgG1κ mAb that binds to CD40 | 03/2018 | 09/2024 |
| NCT03690869 | REGN2810 in Pediatric Patients With Relapsed, Refractory Solid, or Central Nervous System (CNS) Tumors and Safety and Efficacy of REGN2810 in Combination With Radiotherapy in Pediatric Patients With Newly Diagnosed or Recurrent Glioma | I/II | Terminated | Cemiplimab +/- Radiation | 09/2018 | 05/2023 |
| NCT02359565 | Pembrolizumab in Treating Younger Patients With Recurrent, Progressive, or Refractory High-Grade Gliomas, Diffuse Intrinsic Pontine Gliomas, Hypermutated Brain Tumors, Ependymoma or Medulloblastoma | I | Recruiting | Pembrolizumab | 05/2015 | 12/2026 |
| NCT01502917 | Convection-Enhanced Delivery of 124I-Omburtamab for Patients With Non-Progressive Diffuse Pontine Gliomas Previously Treated With External Beam Radiation Therapy | I | Completed | Radioactive iodine-labeled monoclonal antibody omburtamab with External Beam Radiotherapy | 12/2011 | 01/2022 |
| NCT04730349 | A Study of Bempegaldesleukin (BEMPEG: NKTR-214) in Combination With Nivolumab in Children, Adolescents and Young Adults With Recurrent or Treatment-resistant Cancer | I/II | Terminated | Nivolumab and: NKTR-214 | 06/2021 | 06/2022 |
| NCT04238819 | A Study of Abemaciclib (LY2835219) in Combination With Other Anti-Cancer Treatments in Children and Young Adult Participants With Solid Tumors, Including Neuroblastoma | I/II | Active - Not Recruiting | Abemaciclib in Combination with Dinutuximab, GM-CSF, Irinotecan, and Temozolomide | 11/2020 | 04/2025 |
| NCT01145170 | Nimotuzumab and Radiotherapy in Pediatric Patients With Glioma | II | Completed | Nimotuzumab and Radiotherapy | 03/2011 | 07/2014 |
| NCT04167618 | 177Lu-DTPA-Omburtamab Radioimmunotherapy for Recurrent or Refractory Medulloblastoma | I/II | Terminated | 177Lu-DTPA-Omburtamab | 09/2021 | 08/2022 |
| NCT04808245* | A MultIceNTER Phase I Peptide VaCcine Trial for the Treatment of H3-Mutated Gliomas | I | Recruiting | H3K27M peptide vaccine with standard Radiotherapy (RT) and Atezolizumab | 02/2023 | 03/2025 |
| NCT03130959 | A Study to Evaluate the Safety and Efficacy of Nivolumab Monotherapy and Nivolumab in Combination With Ipilimumab in Pediatric Participants With High Grade Primary Central Nervous System (CNS) Malignancies | II | Completed | Nivolumab and Ipilimumab | 06/2017 | 03/2020 |
| NCT02793466 | Durvalumab in Pediatric and Adolescent Patients | I | Completed | Durvalumab | 07/2016 | 11/2022 |
| NCT06896110* | Intrathecal Azacitidine and Nivolumab in Patients With Recurrent High-grade Glioma | I | Active - Not Yet Recruiting | Intrathecal Nivolumab and Azacitadine | 05/2025 | 03/2027 |
| NCT06712875* | MAPK Inhibition Combined with Anti-PD1 Therapy for BRAF-altered Pediatric Gliomas | I/II | Recruiting | Trametinib and Nivolumab | 03/2025 | 06/2029 |
| NCT07017816 | A Phase 0/1 Study of cDNA for TP53, Checkpoint Inhibition and Radiation in Children With Recurrent, Progressive or Refractory CNS Malignancies. | I | Not Yet Recruiting | Nivolumab + SGT53 | 07/2025 | 05/2028 |
| NCT06466798 | Fourth Ventricular Administration of Immune Checkpoint Inhibitor (Nivolumab) and Methotrexate or 5-Azacytidine for Recurrent Medulloblastoma, Ependymoma, and Other CNS Malignancies | I | Recruiting | Nivolumab, Methotrexate, 5-Azacytidine | 07/2024 | 07/2027 |
| NCT06325683 | Anti-Lag-3 (Relatlimab) and Anti-PD-1 Blockade (Nivolumab) Versus Standard of Care (Lomustine) for the Treatment of Patients With Recurrent Glioblastoma | II | Recruiting | Nivolumab, Relatlimab | 11/2024 | 07/2028 |
| NCT05704647 | Phase II Study of Nivolumab in Combination With Relatlimab in Patients With Active Melanoma Brain Metastases | II | Recruiting | (Relatlimab-Nivolumab FDC), Nivolumab, Relatlimab | 02/2023 | 07/2028 |
| NCT04323046 | Immunotherapy Before and After Surgery for Treatment of Recurrent or Progressive High Grade Glioma in Children and Young Adults | I | Recruiting | Nivolumab | 10/2020 | 03/2029 |

Supplementary Table 2. Recent Immune Checkpoint Blockade Trials in DIPG/DMG

| NCT Number | Study Title | Phases | Study Status | Interventions | Start Date | End Date |
| --- | --- | --- | --- | --- | --- | --- |
| NCT04196413 | GD2 CAR T Cells in Diffuse Intrinsic Pontine Gliomas(DIPG) & Spinal Diffuse Midline Glioma(DMG) | I | Recruiting | GD2 CAR T cells \| Fludarabine \| Cyclophosphamide | 06/2020 | 07/2043 |
| NCT04185038 | Study of B7-H3-Specific CAR T Cell Locoregional Immunotherapy for Diffuse Intrinsic Pontine Glioma/Diffuse Midline Glioma and Recurrent or Refractory Pediatric Central Nervous System Tumors | I | Recruiting | B7H3-specific CAR T cells | 12/2019 | 05/2041 |
| NCT05298995 | GD2-CAR T Cells for Pediatric Brain Tumours | I | Recruiting | GD2-CART01 (iC9-GD2-CAR T-cells) | 11/2023 | 11/2038 |
| NCT05768880 | Study of B7-H3, EGFR806, HER2, And IL13-Zetakine (Quad) CAR T Cell Locoregional Immunotherapy For Pediatric Diffuse Intrinsic Pontine Glioma, Diffuse Midline Glioma, And Recurrent Or Refractory Central Nervous System Tumors | I | Recruiting | SC-CAR4BRAIN | 05/2023 | 12/2043 |
| NCT02840123 | Safety Study of DIPG Treatment With Autologous Dendritic Cells Pulsed With Lysated Allegenic Tumor Lines | I | Unknown | Autologous Dendritic Cells Pulsed With Lysated Allegenic Tumor Lines | 06/2016 | 03/2019 |
| NCT06221553 | Safety and Efficacy of Loco-regional B7H3 IL-7Ra CAR T Cell in DIPG | I | Recruiting | B7H3 specific CAR T cell with IL-7Ra Signaling Domain | 03/2024 | 09/2026 |
| NCT03396575 | Brain Stem Gliomas Treated With Adoptive Cellular Therapy During Focal Radiotherapy Recovery Alone or With Dose-intensified Temozolomide (Phase I) | I | Active - Not Recruiting | TTRNA-DC vaccines with GM-CSF and TTRNA-xALT plus Td vaccine with Autologous Hematopoietic Stem cells (HSCs) | 07/2018 | 06/2025 |
| NCT05544526 | CAR T Cells to Target GD2 for DMG | I | Recruiting | GD2 CAR T cells | 08/2023 | 12/2039 |
| NCT04837547 | PEACH TRIAL- Precision Medicine and Adoptive Cellular Therapy | I | Recruiting | Tumor-specific ex vivo expanded autologous lymphocyte transfer | 09/2021 | 09/2030 |
| NCT06396481 | Clinical Study of Allogeneic VŒ≥9VŒ¥2 T Cells in the Treatment of Brain Malignant Glioma | I | Not-Yet-Recruiting | VŒ≥9VŒ¥2 T cell | 04/2024 | 12/2027 |
| NCT05835687 | Loc3CAR: Locoregional Delivery of B7-H3-CAR T Cells for Pediatric Patients With Primary CNS Tumors | I | Recruiting | B7-H3-CAR T cells | 04/2023 | 03/2028 |
| NCT04099797 | C7R-GD2.CAR T Cells for Patients with GD2-expressing Brain Tumors (GAIL-B) | I | Recruiting | C7R-GD2.CAR T Cells | 02/2020 | 02/2039 |
| NCT05478837 | Genetically Modified Cells (KIND T Cells) for the Treatment of HLA-A*0201-Positive Patients With H3.3K27M-Mutated Glioma | I | Recruiting | Autologous Anti-H3.3K27M TCR-expressing T-cells \|Cyclophosphamide \|Fludarabine, | 07/2023 | 08/2029 |
| NCT00005952 | Temozolomide Plus Peripheral Stem Cell Transplantation in Treating Children With Newly Diagnosed Malignant Glioma or Recurrent CNS or Other Solid Tumors | I/II | Completed | Peripheral Stem Cell Transplantation \|Temozolomide | 08/2000 | 11/2005 |
| NCT00078988 | High-Dose Chemotherapy Plus Autologous Stem Cell Transplantation Compared With Intermediate-Dose Chemotherapy Plus Autologous Stem Cell Transplantation With or Without Isotretinoin in Treating Young Patients With Recurrent High-Grade Gliomas | III | Completed | Autologous Stem Cell Transplantation \| Chemotherapy | 10/2004 | 09/2006 |
| NCT00053118 | Chemotherapy and Stem Cell Transplantation in Treating Children With Central Nervous System Cancer | I | Completed | Peripheral blood stem cell transplantation \| Chemotherapy | 03/2002 | 07/2004 |
| NCT03500991 | HER2-specific CAR T Cell Locoregional Immunotherapy for HER2-positive Recurrent/Refractory Pediatric CNS Tumors | I | Active - Not Recruiting | HER2-specific CAR T cell | 07/2018 | 07/2039 |
| NCT06193759 | Immunotherapy for Malignant Pediatric Brain Tumors Employing Adoptive Cellular Therapy (IMPACT) | I | Recruiting | Multi-tumor antigen specific cytotoxic T lymphocytes (TSA-T) directed against personalized tumor-specific antigens (TSA) | 09/2024 | 12/2032 |
| NCT04758533* | Clinical Trial to Assess the Safety and Efficacy of AloCELYVIR With Newly Diagnosed Diffuse Intrinsic Pontine Glioma (DIPG) in Combination With Radiotherapy or Medulloblastoma in Monotherapy | I/II | Active - Not Recruiting | Bone Marrow-derived Allogenic Mesenchymal Stem Cells Infected with an Oncolytic Adenovirus | 04/2021 | 04/2026 |
| NCT03334305* | Adoptive Cellular Therapy in Pediatric Patients With High-grade Gliomas | I | Active - Not Recruiting | Dose-intensified TMZ with TTRNA-DC vaccines with GM-CSF and TTRNA-xALT plus Td vaccine +/- Autologous Hematopoietic Stem cells (HSCs) | 05/2018 | 05/2028 |
| NCT04510051 | CAR T Cells After Lymphodepletion for the Treatment of IL13RŒ±2 Positive Recurrent or Refractory Brain Tumors in Children | I | Recruiting | IL13[EQ]BBzeta/CD19t+ Tn/mem cells | 12/2020 | 08/2025 |
| NCT04003649 | IL13Ra2-CAR T Cells with or Without Nivolumab and Ipilimumab in Treating Patients with GBM | I | Recruiting | IL13Ra2-CAR T Cells +/- Nivolumab and Ipilimumab | 12/2019 | 03/2025 |
| NCT03638167 | EGFR806-specific CAR T Cell Locoregional Immunotherapy for EGFR-positive Recurrent or Refractory Pediatric CNS Tumors | I | Completed | EGFR806-specific CAR T cell | 03/2019 | 12/2023 |
| NCT02442297 | T Cells Expressing HER2-specific Chimeric Antigen Receptors(CAR) for Patients With HER2-Positive CNS Tumors | I | Active Not Recruiting | HER2-specific T cells | 04/2016 | 04/2037 |
| NCT02209376 | Autologous T Cells Redirected to EGFRVIII-With a Chimeric Antigen Receptor in Patients With EGFRVIII+ Glioblastoma | I | Terminated | CART-EGFRvIII T cells | 11/2014 | 04/2018 |
| NCT02208362 | Genetically Modified T-cells in Treating Patients With Recurrent or Refractory Malignant Glioma | I | Active Not Recruiting | IL13Ra2-CAR/CD19t+ Tcm | 05/2015 | 12/2024 |
| NCT01454596 | CAR T Cell Receptor Immunotherapy Targeting EGFRvIII for Patients With Malignant Gliomas Expressing EGFRvIII | I/II | Completed | Ex vivo tumorreactive, CAR gene-transduced peripheral blood mononuclear cells (PBMC) \| IV aldesleukin \|Cyclophosphamide \| Fludarabine | 05/2012 | 01/2019 |
| NCT06946680 | IL-8 Receptor-modified CD70 CAR T Cell Therapy in CD70+ Pediatric High-grade Glioma (HGG) | I | Recruiting | Ex-Vivo expanded autologous IL-8 receptor (CXCR2) modified CD70 CAR-T cells | 05/2025 | 12/2045 |
| NCT07087002 | GPC2-CAR T Cell Therapy for Relapsed or Refractory Medulloblastoma in Children and Young Adults | I | Recruiting | GPC2-CAR T cells | 08/2025 | 08/2027 |
| NCT07031765 | Peds CHAMP1ON - Hematopoietic Stem Cell And Monoclonal Antibody PD-1 Blockade for RecurreNt Pediatric High-Grade Glioma | I | Recruiting | Hematopoietic Stem Cell And Nivolumab | 07/2025 | 12/2032 |
| NCT05887882 | Intra-Tumoral Injections of Natural Killer Cells for Recurrent Malignant Pediatric Brain Tumors | I | Recruiting | Universal Donor (UD) Transforming growth factor beta imprinting (TGFŒ≤i) Natural Killer (NK) Cells | 05/2024 | 12/2027 |
| NCT06514898* | Adoptive T Cell Therapy, DC Vaccines, and Hematopoietic Stem Cells Combined With Immune checkPOINT Blockade in Patients With Medulloblastoma | I | Recruiting | TTRNA-DC vaccines with GM-CSF, TTRNA-xALT, Td vaccine, autologous HSCs, Pembrolizumab | 05/2025 | 12/2028 |

Supplementary Table 3. Recent Adoptive Cell Transfer Trials in DIPG/DMG

| NCT Number | Study Title | Phases | Study Status | Interventions | Start Date | End Date |
| --- | --- | --- | --- | --- | --- | --- |
| NCT05096481 | PEP-CMV Vaccine Targeting CMV Antigen to Treat Newly Diagnosed Pediatric HGG and DIPG and Recurrent Medulloblastoma | II | Recruiting | PEP-CMV Vaccine | 07/2024 | 06/2030 |
| NCT02960230 | H3.3K27M Peptide Vaccine With Nivolumab for Children With Newly Diagnosed DIPG and Other Gliomas | I/II | Completed | H3.3K27M Peptide Vaccine \| Nivolumab | 11/2016 | 12/2023 |
| NCT06305910 | CD200AR-L and Allogeneic Tumor Lysate Vaccine Immunotherapy for Recurrent HGG and Newly Diagnosed DMG/DIPG in Children and Young Adults | I | Recruiting | CD200AR-L and Allogeneic Tumor Lysate Vaccine Immunotherapy | 03/2024 | 01/2027 |
| NCT04911621 | Adjuvant Dendritic Cell Immunotherapy for Pediatric Patients With High-grade Glioma or Diffuse Intrinsic Pontine Glioma | I/II | Active - Not Recruiting | Dendritic cell vaccination \| Temozolomide | 09/2021 | 06/2027 |
| NCT06639607 | PEP-CMV + Nivolumab for Newly Diagnosed Diffuse Midline Glioma/High-grade Glioma and Recurrent Diffuse Midline Glioma/High-grade Glioma, Medulloblastoma, and Ependymoma | I/II | Not-Yet-Recruiting | PEP-CMV Vaccine\| Nivolumab | 01/2025 | 01/2042 |
| NCT03914768 | Immune Modulatory DC Vaccine Against Brain Tumor | I | Unknown | DC Vaccine | 03/2019 | 12/2022 |
| NCT04978727 | A Pilot Study of SurVaxM in Children Progressive or Relapsed Medulloblastoma, High Grade Glioma, Ependymoma and Newly Diagnosed Diffuse Intrinsic Pontine Glioma | I | Recruiting | SurVaxM | 07/2022 | 06/2028 |
| NCT01130077 | A Pilot Study of Glioma Associated Antigen Vaccines in Conjunction With Poly-ICLC in Pediatric Gliomas | I | Active - Not Recruiting | HLA-A2 restricted glioma antigen peptides vaccine and Poly-ICLC | 02/2009 | 12/2025 |
| NCT04749641 | Neoantigen Vaccine Therapy Against H3.3-K27M Diffuse Intrinsic Pontine Glioma | I | Recruiting | Histone H3.3-K27M Neoantigen Vaccine Therapy | 03/2021 | 12/2024 |
| NCT01400672 | Imiquimod/Brain Tumor Initiating Cell (BTIC) Vaccine in Brain Stem Glioma | I | Terminated | Tumor Lysate Vaccine\| Imiquimod \|Radiation therapy | 07/2012 | 10/2018 |
| NCT01808820 | Dendritic Cell (DC) Vaccine for Malignant Glioma and Glioblastoma | I | Completed | Dendritic Cell Vaccine | 08/2013 | 07/2022 |
| NCT04573140 | A Study of RNA-lipid Particle (RNA-LP) Vaccines for Newly Diagnosed Pediatric High-Grade Gliomas (pHGG) and Adult Glioblastoma (GBM) | I/II | Recruiting | RNA-lipid Particle Vaccine | 10/2021 | 07/2030 |
| NCT01902771 | Dendritic Cell Vaccine Therapy With In Situ Maturation in Pediatric Brain Tumors | I | Terminated | Dendritic Cell Vaccine | 09/2013 | 01/2017 |
| NCT02332889 | Phase I/II: Decitabine/Vaccine Therapy in Relapsed/Refractory Pediatric High Grade Gliomas/Medulloblastomas/CNS PNETs | I/II | Terminated | Autologous Dendritic Cells Vaccine\| Decitabine\| Hiltonol | 04/2015 | 07/2016 |
| NCT03615404 | Cytomegalovirus (CMV) RNA-Pulsed Dendritic Cells for Pediatric Patients and Young Adults With WHO Grade IV Glioma, Recurrent Malignant Glioma, or Recurrent Medulloblastoma | I | Completed | Cytomegalovirus (CMV) RNA-Pulsed Dendritic Cells Vaccine | 10/2018 | 07/2020 |
| NCT02722512 | Trial of Heat Shock Protein Peptide Complex-96 (HSPPC-96) Vaccine | I | Terminated | Heat Shock Protein Peptide Complex-96 (HSPPC-96) Vaccine and Radiation Therapy | 07/2016 | 11/2019 |
| NCT03299309 | PEP-CMV in Recurrent MEdulloblastoma/Malignant Glioma | I | Active - Not Recruiting | PEP-CMV Vaccine | 06/2018 | 04/2025 |
| NCT00107185 | Vaccine Therapy in Treating Young Patients Who Are Undergoing Surgery for Malignant Glioma | I | Completed | Therapeutic autologous dendritic cells | 01/2005 | 03/2010 |
| NCT03334305* | Adoptive Cellular Therapy in Pediatric Patients With High-grade Gliomas | I | Active - Not Recruiting | Dose-intensified TMZ with TTRNA-DC vaccines with GM-CSF and TTRNA-xALT plus Td vaccine +/- Autologous Hematopoietic Stem cells (HSCs) | 05/2018 | 05/2028 |
| NCT04808245* | A MultIceNTER Phase I Peptide VaCcine Trial for the Treatment of H3-Mutated Gliomas | I | Recruiting | H3K27M peptide vaccine \| Radiotherapy \| Atezolizumab | 02/2023 | 03/2025 |
| NCT01795313 | Immunotherapy for Recurrent Ependymomas in Children Using Tumor Antigen Peptides with Imiquimod | I | Recruiting | HLA-A2 restricted synthetic tumor antigen Vaccine | 08/2012 | 12/2026 |

Supplementary Table 4. Recent Vaccine Trials in DIPG/DMG

| NCT Number | Study Title | Phases | Study Status | Interventions | Start Date | End Date |
| --- | --- | --- | --- | --- | --- | --- |
| NCT03178032 | Oncolytic Adenovirus, DNX-2401, for Naive Diffuse Intrinsic Pontine Gliomas | I | Completed | Oncolytic Adenovirus, DNX-2401 | 05/2017 | 01/2021 |
| NCT05717712 | Oncolytic Virus Ad-TD-nsIL12 for Primary Pediatric Diffuse Intrinsic Pontine Glioma | I | Recruiting | Oncolytic Virus Ad-TD-nsIL12 | 01/2023 | 01/2028 |
| NCT05717699 | Oncolytic Virus Ad-TD-nsIL12 for Progressive Pediatric Diffuse Intrinsic Pontine Glioma | I | Recruiting | Oncolytic Virus Ad-TD-nsIL12 | 01/2023 | 01/2028 |
| NCT02444546 | Wild-Type Reovirus in Combination With Sargramostim in Treating Younger Patients With High-Grade Relapsed or Refractory Brain Tumors | I | Completed | Wild-Type Reovirus in Combination With Sargramostim | 06/2015 | 11/2022 |
| NCT03043391 | Phase 1b Study PVSRIPO for Recurrent Malignant Glioma in Children | I | Completed | Polio/Rhinovirus Recombinant (PVSRIPO) | 11/2017 | 03/2022 |
| NCT02457845 | HSV G207 Alone or With a Single Radiation Dose in Children With Progressive or Recurrent Supratentorial Brain Tumors | I | Completed | HSV G207 | 05/2016 | 01/2024 |
| NCT03911388 | HSV G207 in Children With Recurrent or Refractory Cerebellar Brain Tumors | I | Active - Not Recruiting | HSV G207 | 09/2019 | 09/2027 |
| NCT04758533* | Clinical Trial to Assess the Safety and Efficacy of AloCELYVIR With Newly Diagnosed Diffuse Intrinsic Pontine Glioma (DIPG) in Combination With Radiotherapy or Medulloblastoma in Monotherapy | I/II | Active - Not Recruiting | Bone Marrow-derived Allogenic Mesenchymal Stem Cells Infected with an Oncolytic Adenovirus | 04/2021 | 04/2026 |
| NCT00805376 | DNX-2401 (Formerly Known as Delta-24-RGD-4C) for Recurrent Malignant Gliomas | I | Completed | DNX-2401 (Delta-24-RGD-4C) | 02/2009 | 02/2015 |
| NCT03072134 | Neural Stem Cell Based Virotherapy of Newly Diagnosed Malignant Glioma | I | Completed | Neural stem cells loaded with an oncolytic adenovirus | 04/2017 | 07/2021 |
| NCT06914479 | Virus-Based Gene Therapy (AdV-HSV1-TK and AdV-Flt3L) in Combination With Valacyclovir for the Treatment of Pediatric and Young Adult Patients With Resectable, Recurrent Primary Malignant Brain Tumors | I | Active – Not Yet Recruiting | AdV-HSV1-TK and AdV-Flt3L | 06/2025 | 06/2030 |
| NCT07076498 | Engineered HSV-1 M032 for the Treatment of Children and Adults With Newly Diagnosed Diffuse Midline Glioma After Standard of Care Radiation | I | Not Yet Recruiting | HSV-1 M032 | 12/2025 | 12/2031 |

Supplementary Table 5. Recent Oncolytic Virus Trials in DIPG/DMG
