# Supplementary material for: Deciphering the tumor microenvironment and role of immunotherapy in diffuse midline glioma: A scoping review
Source: Neuro Oncol. 2026 Feb 2;28(4):829–46. doi: 10.1093/neuonc/noag014 (PMC13128486; doi:10.1093/neuonc/noag014)
Supplement: noag014_Supplementary_Data [file noag014_supplementary_data.zip › Supplement 1.docx]

CHALLENGES FROM TARGETING GENOMIC ABERRATIONS AND AFFECTED PATHWAYS IN DMG

Though the underlying mechanism of each DMG subtype overlaps substantially, subtle differences have been reported regarding co-occurring somatic mutations, locality, and median overall survival.^1^ The genomic and transcriptomic landscape of DMG has been comprehensively characterized and similar to many adult cancers including adult glioblastoma, DMG harbors inter- as well as intra-tumoral heterogeneity.^2, 3^ Temporally, it has been determined that the H3K27M mutation arises first and is subsequently associated with specific, obligate partners involving mutations in the TP53 cell cycle or specific growth factor pathways.^4^ Nikbakht and colleagues used evolutionary analysis to identify four primary driver gene combinations in DMG: H3K27M/TP53 and/or PPM1D, H3K27M/ACR1, and H3K27M/PIK3R1. Of note, each autopsied brain sample consistently exhibits the same primary driver mutation partnership, a notable contrast to adult glioblastoma, which typically contains multiple distinct evolutionary histories within different tumor foci.^5^ Three categories of mutations have been postulated in DMG: (1) main driver mutations that initiate and continue tumorigenesis; (2) accessory driver mutations that promote and accelerate tumor growth, but are not essential; (3) passenger mutations, which do not affect the tumor. Other than H3K27M, the main classified drivers are TP53, PPM1D, ACVR1, and PIK3R1. Further, they identified PIK3CA and PTEN as sub-clonal and accessory driver mutations. Finally, the authors hypothesized that the partner mutation is selected for its ability to maximize mutant H3 levels. Canonical H3 variants (H3.1, H3.2) are the most abundant histones in cells and require cell division (S-phase) for synthesis while non-canonical H3.3 variants comprise only around 5% of all H3, but are present throughout the cell cycle. H3.1K27M mutations in cell cycle-dependent canonical histones tend to co-occur with alterations in ACVR1, a growth factor that promotes cell division. In contrast, H3.3K27M mutations are primarily associated with mutations in the TP53 pathway, which may allow for the evasion of cell death and senescence and provide the cell with the time needed to reshape the epigenome and drive tumor formation.

The characterization of somatic mutations in DMG provides important insights into the affected oncogenic pathways and reveals potential therapeutic vulnerabilities. Unfortunately, despite the development of targeted therapies against somatic mutations and affected pathways in DMG, the vast majority have either failed due to the BBB (blood brain barrier), toxicities or are still in early-stage clinical trials.^3^ More recent studies are exploring combinatorial regimens, but the maximum tolerated doses and efficacy are yet to be determined.

As highlighted in the previous section, DMG tumors exhibit high levels of both inter- and intra-tumoral clonal heterogeneity, with multiple somatic subclones co-existing in the spatial and temporal dimensions.^22, 23^ High-throughput drug screening campaigns with molecular analysis have been employed to assess drug sensitivities in a plethora of DMG models, which aim to correlate genomic influences with potential treatment strategies.^24, 25^ Subclones are crucial to tumorigenesis, enhancing proliferation, invasion, and oncogenic signaling, particularly in response to CNS-active therapies.^21^ Thus, given the complexity of the clonal cancer genome, a one-size-fits-all targeted approach is likely destined for failure as tumors typically harbor multiple mutations with unclear significance, which may complicate the identification of oncogenic drivers.

Transplanting or co-culturing DMG subclones with less mobile colonies has been shown to augment invasiveness, which suggests a cooperative role in tumor cell dissemination.^23^ While targeting specific subclone mutations may provide temporary benefits, the genomic diversity of subclones will ultimately limit the long-term efficacy of targeted approaches.^26^ Furthermore, the tumor microenvironment (TME) and non-genomic factors such as corticosteroid therapy, growth factors, and stress hormones have been found to contribute to increased invasiveness and intertumoral heterogeneity, complicating the treatment efforts. Moving forward, it is critical that

therapeutic regimens for DMG consider not only the characteristics of the tumor itself but also the microenvironment that encapsulates and protects it.

References:

1. Persson ML, Douglas AM, Alvaro F, Faridi P, Larsen MR, Alonso MM, Vitanza NA, Dun MD. The intrinsic and microenvironmental features of diffuse midline glioma: Implications for the development of effective immunotherapeutic treatment strategies. Neuro Oncol. 2022;24(9):1408-22. doi: 10.1093/neuonc/noac117. PubMed PMID: 35481923; PMCID: PMC9435509.

2. Mackay A, Burford A, Carvalho D, Izquierdo E, Fazal-Salom J, Taylor KR, Bjerke L, Clarke M, Vinci M, Nandhabalan M, Temelso S, Popov S, Molinari V, Raman P, Waanders AJ, Han HJ, Gupta S, Marshall L, Zacharoulis S, Vaidya S, Mandeville HC, Bridges LR, Martin AJ, Al-Sarraj S, Chandler C, Ng HK, Li X, Mu K, Trabelsi S, Brahim DH, Kisljakov AN, Konovalov DM, Moore AS, Carcaboso AM, Sunol M, de Torres C, Cruz O, Mora J, Shats LI, Stavale JN, Bidinotto LT, Reis RM, Entz-Werle N, Farrell M, Cryan J, Crimmins D, Caird J, Pears J, Monje M, Debily MA, Castel D, Grill J, Hawkins C, Nikbakht H, Jabado N, Baker SJ, Pfister SM, Jones DTW, Fouladi M, von Bueren AO, Baudis M, Resnick A, Jones C. Integrated Molecular Meta-Analysis of 1,000 Pediatric High-Grade and Diffuse Intrinsic Pontine Glioma. Cancer Cell. 2017;32(4):520-37.e5. Epub 20170928. doi: 10.1016/j.ccell.2017.08.017. PubMed PMID: 28966033; PMCID: PMC5637314.

3. Findlay IJ, De Iuliis GN, Duchatel RJ, Jackson ER, Vitanza NA, Cain JE, Waszak SM, Dun MD. Pharmaco-proteogenomic profiling of pediatric diffuse midline glioma to inform future treatment strategies. Oncogene. 2022;41(4):461-75. doi: 10.1038/s41388-021-02102-y.

4. Nikbakht H, Panditharatna E, Mikael LG, Li R, Gayden T, Osmond M, Ho C-Y, Kambhampati M, Hwang EI, Faury D, Siu A, Papillon-Cavanagh S, Bechet D, Ligon KL, Ellezam B, Ingram WJ, Stinson C, Moore AS, Warren KE, Karamchandani J, Packer RJ, Jabado N, Majewski J, Nazarian J. Spatial and temporal homogeneity of driver mutations in diffuse intrinsic pontine glioma. Nature Communications. 2016;7(1):11185. doi: 10.1038/ncomms11185.

5. Eisenbarth D, Wang YA. Glioblastoma heterogeneity at single cell resolution. Oncogene. 2023;42(27):2155-65. doi: 10.1038/s41388-023-02738-y.
