# Supplementary material for: Deciphering the tumor microenvironment and role of immunotherapy in diffuse midline glioma: A scoping review
Source: Neuro Oncol. 2026 Feb 2;28(4):829–46. doi: 10.1093/neuonc/noag014 (PMC13128486; doi:10.1093/neuonc/noag014)
Supplement: noag014_Supplementary_Data [file noag014_supplementary_data.zip › Supplement 2.docx]

Recent Advancements from Single-cell, Spatial Analyses, and Preclinical Models

Despite some debate in the field,^1^ multiple lines of evidence suggest that early oligodendroglial lineage precursors are the likely cells of origin for DMGs.^2-10^ Careful histological analyses of the human brainstem across early post-natal development identified an early oligodendroglial precursor cell population (pre-OPCs) prevalent at the same time and place that DIPG typically arises.^2^ Immunohistochemical analyses have suggested that the pons may be susceptible to tumor formation throughout childhood as a vimentin-positive, nestin-positive cell population persists in the pons, and a majority of DIPGs express SOX2 and OLIG2, markers of stemness and oligodendrocyte development respectively. ^3-5^ Subsequent superenhancer analyses of patient DMG samples further indicated origins in an early oligodendrocyte precursor cell (OPC or pre-OPC), including that OPCs recapitulate the active chromatin state of DMG better than neural precursors (NPCs).^6, 8^ Early single-cell work led by Filbin and colleagues further characterized the cellular architecture of DMG through single-cell RNA sequencing (scRNA-seq) on 3321 cells from six primary H3K27M-glioma and matched models.^11^ They found that H3K27M-glioma predominantly consist of malignant cells that resemble oligodendrocyte precursor cells (OPC-like), whereas the more differentiated cells of astrocytic differentiation (AC-like) and oligodendrocytic differentiation (OC-like) are the minority. Moreover, OPC-like cells exhibit greater proliferation, tumor-propagating potential, and presumably can either self-renew or give rise to their more differentiated counterparts. Subsequent work further showed that the H3K27M mutation directly impacts cellular differentiation by preventing complete differentiation along the glial lineages.^12^ A landmark study in 2022 performed multi-omic profiling of 50 adult, adolescent, and pediatric patients with H3K27M DMG, covering a representative clinic-molecular range.^10^ Unlike other high-grade gliomas such as adult glioblastoma, no neuronal lineage tumor cells were detected in DMG tumors. Thus, it was postulated that the K27M mutation has a direct cell-intrinsic effect to tilt tumor cells towards a glial/OPC-like identity regardless of age or anatomical location. As a function of regional specificity, this study also suggested that pontine DMGs had more immature OPC-like cells (termed pre-OPC-like cells) compared to their thalamic counterparts. Utilizing a hybridization-based in situ sequencing (HybISS) approach, this work was one of the first to assess the single-cell spatial architecture of DMG.^10^ Surprisingly, the more differentiated AC-like cells were found to constitute most of the cell population in situ, which contradicts the previous findings of OPC-like cell predominance observed by scRNA-seq. This discrepancy was speculated to arise due to technical and biological causes specific to scRNA-seq processing.

Lastly, the authors observed that glioma-associated myeloid cells (GAMs) made up the largest population of nonmalignant cells within their scRNAseq dataset^10^, consistent with previous histological studies demonstrating GAMs as the predominant immune cell present in DMGs.^13^ Though overall GAM proportions were consistent between tumors from adult and children, pediatric DMGs displayed higher rates for brain-resident microglia while adult DMGs were enriched with monocyte-derived macrophages. The authors highlight evidence that GAMs secrete ligands, such as OSM and OSMR, contribute to the mesenchymal cell state in adult glioblastoma, and were also found to promote MES-like tumor cell states in H3-K27M DMG-associated GAMs as age increases.^14-16^ This marked one of the first instances where microenvironmental factors were found to distinctly shape tumor cellular states in DMG, which has propelled further investigation into the role of the myeloid population in H3K27M tumors.

A series of breakthrough papers in 2024 shed light on the TME of DMG, specifically the myeloid-derived subpopulations, which has important clinical implications for future immunotherapies. Levine and colleagues developed a dataset with 1382 pediatric brain tumor samples that detailed immune-oncology gene expression as well as clinical and molecular annotations.^17^ They repeatedly utilized an 18-gene tumor inflammation signature (TIS) that has been previously validated as a biomarker for the ICI response. Their results showed that both LGG and HGG have evidence of high immune activation and, in contrast with previous studies, DMGs have a substantially higher TIS score compared to non-tumor brain and hemispheric HGG. Given the novelty of this finding, they verified with immunohistochemistry (IHC) and showed high expression of several immune checkpoints on a subset of DMG samples including those with currently available FDA-approved drugs (PD-1, PD-L1) and drugs under development (LAG3, TIGIT). They attributed this newly discovered feature of DMG to the fact that their analysis included substantially more DMG samples than prior ones with 59 in-house and 99 PBTA samples, compared to only 13^13^ and 9^18^ samples in previous publications. They also studied TIS in the context of mismatch repair deficient (MMRD) HGGs where they assessed the utility of TIS compared to tumor mutational burden (TMB) for its use as a biomarker for predicting immunotherapy treatment response. Although they found no relationship between TIS and TMB, they demonstrated that each metric provides complementary information. Whereas TIS may be a measure of immune activation, TMB indicates neo-antigenicity. They observed that the best outcomes for recurrent HGG patients treated with ICI occurred when both TIS and TMB were elevated, with several patients achieving progression free survival of 3 years or longer. On the other hand, the worst outcomes were observed for patients with low TIS levels regardless of their TMB status. Thus, DMG and other pHGG patients with both high TMB and high TIS may be candidates for radiation- and chemotherapy-free treatment, given their excellent response to immunotherapy.^19^

Andrade and colleagues sought to further elucidate unanswered questions about the immune infiltrate of DMG by comprehensively combining single-cell transcriptomic and spatial proteomic by imaging mass cytometry (IMC) approaches in patient samples and multiple murine models.^20^ They found that pHGG samples are universally infiltrated by heterogeneous populations of myeloid cells from resident microglia and newly recruited infiltrating macrophages, modest T cell infiltration, and high expression of select immune checkpoint markers on immune and tumor cells. While each type of glioma had a distinct myeloid population profile, H3K27M gliomas exhibited the lowest proportion of lymphoid cells and an absence of activation signatures in K27M-derived microglia. In fact, K27M-derived microglia had a significantly upregulated immunosuppressive signature with upregulation in genes such as *CXC3R1*, *KLF2*, *TGFB1* as well as higher levels of TIM3, Gal-9, and CD86. Thus, highlighting the potential role of myeloid cells in promoting the immunosuppressive TME. To further understand the evolution of the DMG TME, they performed *in vivo* serial engraftments in a syngeneic H3K27M murine model.^20^ Tumors were dissociated and transplanted into new hosts upon signs of tumor burden. With each engraftment, tumor growth accelerated, survival decreased, and the immune infiltration became dominated by myeloid cells, resulting in reduced immune diversity by the third engraftment, which closely resembled that of the human H3K27M TME. The recruited myeloid cells included both resident macrophages and bone marrow-derived macrophages (BMDMs), demonstrating active recruitment to the tumors. Given the failure of lymphocyte-targeting therapies in pHGGs, the authors investigated whether targeting myeloid infiltrates could impact tumor growth, either alone or in combination with ICIs (anti-PD-1).^20^ They focused on CSF1R, a receptor kinase essential for the survival and proliferation of microglia, peripheral macrophages, and infiltrating blood myeloid cells. Targeting CSF1R alone showed no effect on tumor growth, similar to anti-PD-1 therapy. However, the combination of CSF1R and anti-PD-1 significantly increased CD3+ T-cell infiltration and extended survival in mice. This suggests that myeloid modulation may enhance T-cell recruitment and could be a promising strategy in combination with ICIs for treating DMG patients.

Ross et al. similarly aimed to better characterize the myeloid populations of DMG, focusing on glioma/tumor-associated microphages (G/TAMs).^21^ Compared to other pHGGs, DMG clinical samples had the highest expression of genes reflective of a greater presence of CD45+ cells, macrophages, and neutrophils. Utilizing scRNA-seq in DMG mouse models, TAMs were found to account for approximately 28% of all cells sequenced and included brain resident microglia and monocyte-derived macrophages (MDMs). H3.3K27M DMGs had the highest percentage of microglia, which obtain disease-associated signatures that downregulate inflammatory signaling and upregulate proliferative signaling, thereby promoting immune escape and tumor growth. Thus, it was concluded that the K27M oncohistone driver mutations shape the immune infiltrate with a more lymphocyte-depleted TME and establish the presence of disease-associated myeloid (DAM) cells that lack immune stimulatory properties.

The authors then asked whether inhibiting TAM infiltration can provide therapeutic benefits in DMG.^21^ First, they genetically targeted *Ccl8* and *Ccl12*, which myeloid cells express as key chemokines and signals through CCR2 and CCR5, by generating *Ccl8/12* double-null mice. While *Ccl12* is expressed by all myeloid cells, it is primarily found in microglia and *Ccl8* is predominantly expressed by infiltrating MDMs. Although a decrease in TAM infiltration was not observed with the double knockout, the microglia transcriptomic signatures were markedly altered toward a more pro-inflammatory state. Subsequently, the TME was changed with an increase in lymphocyte infiltration and a survival benefit dependent on CD4+ T cell depletion (likely CD4+ T-regs). Additionally, the authors identified a pharmacologic approach to target TAM infiltration in H3K27M DMGs. As opposed to their findings in the genetically modified mice, dual pharmacologic inhibition of CCR1 and CCR5 significantly reduced MDM and microglial populations in the TME. Moreover, there was an increase in CD8+ T cell infiltration and an increase in median survival from 25 to 29.5 weeks, which was comparable to the standard-of-care RT. No changes in myeloid cell numbers in the blood were observed, demonstrating the translatability of systemic, pharmacologic inhibition of myeloid cell infiltration.^21^ Potentially, this protocol can be seamlessly adopted into therapeutic regimens for humans and also combined with other treatment modalities for synergistic effects.

Finally, Damodharan and colleagues recently published their work using the Nanostring GeoMx Digital Spatial Profiling and Illumina sequencing platforms to study the spatial transcriptomic and proteomic landscape in H3K27-altered DMG biopsy samples.^22^ Interestingly, one of their key findings was the limited fidelity between overall transcriptome and proteome in DMG.

While previous studies have suggested that differentially expressed mRNA may be a reliable readout of true protein synthesis,^23^ this has not been widely tested in the context of brain malignancies, especially in the pediatric population. Specifically, they found several common therapeutic targets to have differing RNA to protein correlation. Some of the genes with the lowest concordance between RNA and protein were EGFR and immune checkpoint markers such as PD-L1 and CTLA-4. This result raises concerns for clinical decision-making solely based on transcriptomics, which may result in false positives as well as false negatives, and highlights the importance of integrating proteomic data to strengthen early-stage clinical drug screening.^22^

References:

1. Haag D, Mack N, Benites Goncalves da Silva P, Statz B, Clark J, Tanabe K, Sharma T, Jäger N, Jones DTW, Kawauchi D, Wernig M, Pfister SM. H3.3-K27M drives neural stem cell-specific gliomagenesis in a human iPSC-derived model. Cancer Cell. 2021;39(3):407-22.e13. Epub 20210204. doi: 10.1016/j.ccell.2021.01.005. PubMed PMID: 33545065.

2. Monje M, Mitra SS, Freret ME, Raveh TB, Kim J, Masek M, Attema JL, Li G, Haddix T, Edwards MSB, Fisher PG, Weissman IL, Rowitch DH, Vogel H, Wong AJ, Beachy PA. Hedgehog-responsive candidate cell of origin for diffuse intrinsic pontine glioma. Proceedings of the National Academy of Sciences. 2011;108(11):4453-8. doi: doi:10.1073/pnas.1101657108.

3. Lindquist RA, Guinto CD, Rodas-Rodriguez JL, Fuentealba LC, Tate MC, Rowitch DH, Alvarez-Buylla A. Identification of proliferative progenitors associated with prominent postnatal growth of the pons. Nat Commun. 2016;7:11628. Epub 20160518. doi: 10.1038/ncomms11628. PubMed PMID: 27188978; PMCID: PMC4873968.

4. Ballester LY, Wang Z, Shandilya S, Miettinen M, Burger PC, Eberhart CG, Rodriguez FJ, Raabe E, Nazarian J, Warren K, Quezado MM. Morphologic characteristics and immunohistochemical profile of diffuse intrinsic pontine gliomas. Am J Surg Pathol. 2013;37(9):1357-64. doi: 10.1097/PAS.0b013e318294e817. PubMed PMID: 24076776; PMCID: PMC3787318.

5. Tate MC, Lindquist RA, Nguyen T, Sanai N, Barkovich AJ, Huang EJ, Rowitch DH, Alvarez-Buylla A. Postnatal growth of the human pons: a morphometric and immunohistochemical analysis. J Comp Neurol. 2015;523(3):449-62. Epub 20141202. doi: 10.1002/cne.23690. PubMed PMID: 25307966; PMCID: PMC4270924.

6. Nagaraja S, Vitanza NA, Woo PJ, Taylor KR, Liu F, Zhang L, Li M, Meng W, Ponnuswami A, Sun W, Ma J, Hulleman E, Swigut T, Wysocka J, Tang Y, Monje M. Transcriptional Dependencies in Diffuse Intrinsic Pontine Glioma. Cancer Cell. 2017;31(5):635-52.e6. doi: 10.1016/j.ccell.2017.03.011.

7. Neftel C, Laffy J, Filbin MG, Hara T, Shore ME, Rahme GJ, Richman AR, Silverbush D, Shaw ML, Hebert CM, Dewitt J, Gritsch S, Perez EM, Gonzalez Castro LN, Lan X, Druck N, Rodman C, Dionne D, Kaplan A, Bertalan MS, Small J, Pelton K, Becker S, Bonal D, Nguyen QD, Servis RL, Fung JM, Mylvaganam R, Mayr L, Gojo J, Haberler C, Geyeregger R, Czech T, Slavc I, Nahed BV, Curry WT, Carter BS, Wakimoto H, Brastianos PK, Batchelor TT, Stemmer-Rachamimov A, Martinez-Lage M, Frosch MP, Stamenkovic I, Riggi N, Rheinbay E, Monje M, Rozenblatt-Rosen O, Cahill DP, Patel AP, Hunter T, Verma IM, Ligon KL, Louis DN, Regev A, Bernstein BE, Tirosh I, Suvà ML. An Integrative Model of Cellular States, Plasticity, and Genetics for Glioblastoma. Cell. 2019;178(4):835-49.e21. Epub 20190718. doi: 10.1016/j.cell.2019.06.024. PubMed PMID: 31327527; PMCID: PMC6703186.

8. Nagaraja S, Quezada MA, Gillespie SM, Arzt M, Lennon JJ, Woo PJ, Hovestadt V, Kambhampati M, Filbin MG, Suva ML, Nazarian J, Monje M. Histone Variant and Cell Context Determine H3K27M Reprogramming of the Enhancer Landscape and Oncogenic State. Mol Cell. 2019;76(6):965-80.e12. Epub 20191003. doi: 10.1016/j.molcel.2019.08.030. PubMed PMID: 31588023; PMCID: PMC7104854.

9. Jessa S, Mohammadnia A, Harutyunyan AS, Hulswit M, Varadharajan S, Lakkis H, Kabir N, Bashardanesh Z, Hébert S, Faury D, Vladoiu MC, Worme S, Coutelier M, Krug B, Faria Andrade A, Pathania M, Bajic A, Weil AG, Ellezam B, Atkinson J, Dudley RWR, Farmer JP, Perreault S, Garcia BA, Larouche V, Blanchette M, Garzia L, Bhaduri A, Ligon KL, Bandopadhayay P, Taylor MD, Mack SC, Jabado N, Kleinman CL. K27M in canonical and noncanonical H3 variants occurs in distinct oligodendroglial cell lineages in brain midline gliomas. Nat Genet. 2022;54(12):1865-80. Epub 20221205. doi: 10.1038/s41588-022-01205-w. PubMed PMID: 36471070; PMCID: PMC9742294.

10. Liu I, Jiang L, Samuelsson ER, Marco Salas S, Beck A, Hack OA, Jeong D, Shaw ML, Englinger B, LaBelle J, Mire HM, Madlener S, Mayr L, Quezada MA, Trissal M, Panditharatna E, Ernst KJ, Vogelzang J, Gatesman TA, Halbert ME, Palova H, Pokorna P, Sterba J, Slaby O, Geyeregger R, Diaz A, Findlay IJ, Dun MD, Resnick A, Suvà ML, Jones DTW, Agnihotri S, Svedlund J, Koschmann C, Haberler C, Czech T, Slavc I, Cotter JA, Ligon KL, Alexandrescu S, Yung WKA, Arrillaga-Romany I, Gojo J, Monje M, Nilsson M, Filbin MG. The landscape of tumor cell states and spatial organization in H3-K27M mutant diffuse midline glioma across age and location. Nature Genetics. 2022;54(12):1881-94. doi: 10.1038/s41588-022-01236-3.

11. Filbin MG, Tirosh I, Hovestadt V, Shaw ML, Escalante LE, Mathewson ND, Neftel C, Frank N, Pelton K, Hebert CM, Haberler C, Yizhak K, Gojo J, Egervari K, Mount C, van Galen P, Bonal DM, Nguyen Q-D, Beck A, Sinai C, Czech T, Dorfer C, Goumnerova L, Lavarino C, Carcaboso AM, Mora J, Mylvaganam R, Luo CC, Peyrl A, Popović M, Azizi A, Batchelor TT, Frosch MP, Martinez-Lage M, Kieran MW, Bandopadhayay P, Beroukhim R, Fritsch G, Getz G, Rozenblatt-Rosen O, Wucherpfennig KW, Louis DN, Monje M, Slavc I, Ligon KL, Golub TR, Regev A, Bernstein BE, Suvà ML. Developmental and oncogenic programs in H3K27M gliomas dissected by single-cell RNA-seq. Science. 2018;360(6386):331-5. doi: doi:10.1126/science.aao4750.

12. Jessa S, Blanchet-Cohen A, Krug B, Vladoiu M, Coutelier M, Faury D, Poreau B, De Jay N, Hébert S, Monlong J, Farmer WT, Donovan LK, Hu Y, McConechy MK, Cavalli FMG, Mikael LG, Ellezam B, Richer M, Allaire A, Weil AG, Atkinson J, Farmer J-P, Dudley RWR, Larouche V, Crevier L, Albrecht S, Filbin MG, Sartelet H, Lutz P-E, Nagy C, Turecki G, Costantino S, Dirks PB, Murai KK, Bourque G, Ragoussis J, Garzia L, Taylor MD, Jabado N, Kleinman CL. Stalled developmental programs at the root of pediatric brain tumors. Nature Genetics. 2019;51(12):1702-13. doi: 10.1038/s41588-019-0531-7.

13. Lin GL, Nagaraja S, Filbin MG, Suvà ML, Vogel H, Monje M. Non-inflammatory tumor microenvironment of diffuse intrinsic pontine glioma. Acta Neuropathol Commun. 2018;6(1):51. Epub 20180628. doi: 10.1186/s40478-018-0553-x. PubMed PMID: 29954445; PMCID: PMC6022714.

14. Hara T, Chanoch-Myers R, Mathewson ND, Myskiw C, Atta L, Bussema L, Eichhorn SW, Greenwald AC, Kinker GS, Rodman C, Gonzalez Castro LN, Wakimoto H, Rozenblatt-Rosen O, Zhuang X, Fan J, Hunter T, Verma IM, Wucherpfennig KW, Regev A, Suvà ML, Tirosh I. Interactions between cancer cells and immune cells drive transitions to mesenchymal-like states in glioblastoma. Cancer Cell. 2021;39(6):779-92.e11. Epub 20210603. doi: 10.1016/j.ccell.2021.05.002. PubMed PMID: 34087162; PMCID: PMC8366750.

15. Schmitt MJ, Company C, Dramaretska Y, Barozzi I, Göhrig A, Kertalli S, Großmann M, Naumann H, Sanchez-Bailon MP, Hulsman D, Glass R, Squatrito M, Serresi M, Gargiulo G. Phenotypic Mapping of Pathologic Cross-Talk between Glioblastoma and Innate Immune Cells by Synthetic Genetic Tracing. Cancer Discov. 2021;11(3):754-77. Epub 20201223. doi: 10.1158/2159-8290.Cd-20-0219. PubMed PMID: 33361384; PMCID: PMC7611210.

16. Sa JK, Chang N, Lee HW, Cho HJ, Ceccarelli M, Cerulo L, Yin J, Kim SS, Caruso FP, Lee M, Kim D, Oh YT, Lee Y, Her NG, Min B, Kim HJ, Jeong DE, Kim HM, Kim H, Chung S, Woo HG, Lee J, Kong DS, Seol HJ, Lee JI, Kim J, Park WY, Wang Q, Sulman EP, Heimberger AB, Lim M, Park JB, Iavarone A, Verhaak RGW, Nam DH. Transcriptional regulatory networks of tumor-associated macrophages that drive malignancy in mesenchymal glioblastoma. Genome Biol. 2020;21(1):216. Epub 20200826. doi: 10.1186/s13059-020-02140-x. PubMed PMID: 32847614; PMCID: PMC7448990.

17. Levine AB, Nobre L, Das A, Milos S, Bianchi V, Johnson M, Fernandez NR, Stengs L, Ryall S, Ku M, Rana M, Laxer B, Sheth J, Sbergio SG, Fedoráková I, Ramaswamy V, Bennett J, Siddaway R, Tabori U, Hawkins C. Immuno-oncologic profiling of pediatric brain tumors reveals major clinical significance of the tumor immune microenvironment. Nat Commun. 2024;15(1):5790. Epub 20240710. doi: 10.1038/s41467-024-49595-1. PubMed PMID: 38987542; PMCID: PMC11237052.

18. Lieberman NAP, DeGolier K, Kovar HM, Davis A, Hoglund V, Stevens J, Winter C, Deutsch G, Furlan SN, Vitanza NA, Leary SES, Crane CA. Characterization of the immune microenvironment of diffuse intrinsic pontine glioma: implications for development of immunotherapy. Neuro Oncol. 2019;21(1):83-94. Epub 2018/09/01. doi: 10.1093/neuonc/noy145. PubMed PMID: 30169876; PMCID: PMC6303470.

19. Larkin T, Das A, Bianchi V, Sudhaman S, Chung J, Alsafwani N, Negm L, Yachnis A, Blatt J, Hawkins C, Bouffet E, Tabori U, Gururangan S. Upfront Adjuvant Immunotherapy of Replication Repair-Deficient Pediatric Glioblastoma With Chemoradiation-Sparing Approach. JCO Precis Oncol. 2021;5:1426-31. doi: 10.1200/po.21.00153. PubMed PMID: 34994637.

20. Andrade AF, Annett A, Karimi E, Topouza DG, Rezanejad M, Liu Y, McNicholas M, Gonzalez Santiago EG, Llivichuzhca-Loja D, Gehlhaar A, Jessa S, De Cola A, Chandarana B, Russo C, Faury D, Danieau G, Puligandla E, Wei Y, Zeinieh M, Wu Q, Hebert S, Juretic N, Nakada EM, Krug B, Larouche V, Weil AG, Dudley RWR, Karamchandani J, Agnihotri S, Quail DF, Ellezam B, Konnikova L, Walsh LA, Pathania M, Kleinman CL, Jabado N. Immune landscape of oncohistone-mutant gliomas reveals diverse myeloid populations and tumor-promoting function. Nat Commun. 2024;15(1):7769. Epub 20240905. doi: 10.1038/s41467-024-52096-w. PubMed PMID: 39237515; PMCID: PMC11377583.

21. Ross JL, Puigdelloses-Vallcorba M, Piñero G, Soni N, Thomason W, DeSisto J, Angione A, Tsankova NM, Castro MG, Schniederjan M, Wadhwani NR, Raju GP, Morgenstern P, Becher OJ, Green AL, Tsankov AM, Hambardzumyan D. Microglia and monocyte-derived macrophages drive progression of pediatric high-grade gliomas and are transcriptionally shaped by histone mutations. Immunity. 2024. doi: 10.1016/j.immuni.2024.09.007.

22. Damodharan S, Shireman JM, Xie E, Distler E, Kendziorski C, Dey M. Transcriptomic and proteomic spatial profiling of pediatric and adult diffuse midline glioma H3 K27-Altered. Sci Rep. 2024;14(1):22668. Epub 20240930. doi: 10.1038/s41598-024-73199-w. PubMed PMID: 39349581; PMCID: PMC11443003.

23. Koussounadis A, Langdon SP, Um IH, Harrison DJ, Smith VA. Relationship between differentially expressed mRNA and mRNA-protein correlations in a xenograft model system. Sci Rep. 2015;5:10775. Epub 20150608. doi: 10.1038/srep10775. PubMed PMID: 26053859; PMCID: PMC4459080.
