# Supplementary material for: Deciphering the tumor microenvironment and role of immunotherapy in diffuse midline glioma: A scoping review
Source: Neuro Oncol. 2026 Feb 2;28(4):829–46. doi: 10.1093/neuonc/noag014 (PMC13128486; doi:10.1093/neuonc/noag014)
Supplement: noag014_Supplementary_Data [file noag014_supplementary_data.zip › Abbreviation.docx]

ABBREVIATION TABLE

| ACT | Adoptive cell transfer |
| --- | --- |
| AMPA | α-amino-3-hydroxy-5-methyl-4-isoxazole propionic acid |
| APC | Antigen presenting cells |
| B7-H3 | B7-homolog 3 protein |
| B7-H3 | B7-Homolog 3 protein |
| B-ALL | B cell lineage acute lymphoblastic leukemia |
| BBB | Blood brain barrier |
| BDNF | Brain-derived neurotrophic factor |
| BMDM | Bone marrow-derived macrophages, CCR2+ populations |
| CAR-T | T-cells genetically modified to express chimeric antigen receptor |
| CED | Convection enhanced delivery |
| CNS | Central Nervous System |
| CRS | Cytokine release syndrome |
| CSF | Cerebrospinal fluid |
| CTLA-4 | Cytotoxic T lymphocyte-associated antigen-4 |
| DAM | Disease-associated myeloid |
| DC | Dendritic cell |
| DIPG/DMG | Diffuse Midline Glioma |
| EGFR | Epidermal growth factor receptor |
| EPSCs | Synaptic excitatory postsynaptic currents |
| FAK | Focal adhesion kinase |
| FUS | Focused ultrasound |
| G/TAM | Glioma/tumor-associated microphage |
| GAM | Glioma-associated myeloid |
| GBM | Adult glioblastoma |
| GD2 | Disialoganglioside GD2 |
| GEMM | Genetically engineered mouse model |
| GRP78 | Glioma mitogen 78 kDa glucose-regulated protein |
| H3.1K27M | Mutation in HIST1H3B/C |
| H3.3K27M | Mutation in H3F3A |
| HER2 | ERBB2 receptor tyrosine kinase |
| HybISS | Hybridization-based in situ sequencing |
| ICANS | Immune effector cell-associated neurotoxicity syndrome |
| ICI | Immune checkpoint inhibitor |
| ICB | Immune checkpoint blockade |
| ICP | Intracranial pressure |
| ICV | Intracerebroventricular |
| IHC | Immunohistochemistry |
| IL | Interleukin |
| IMC | Imaging mass cytometry |
| IUE | In utero electroporation |
| IV | Intravenous |
| LDT | Laterodorsal tegmentum nucleus |
| MDM | Monocyte-derived macrophage |
| MMRD | Mismatch repair deficient |
| MSC | Mesenchymal stem cell |
| NLGN3 | Neuroligin 3 |
| oHSV | Oncolytic herpes virus |
| OPC | Oligodendrocyte precursor cells |
| OS | Overall Survival |
| OSM | Oncostatin M |
| OT/OT | On-tumor/off-target |
| OV | Oncolytic virus |
| PD-1 | Programmed cell death protein-1 |
| PD-L1 | Programmed cell death ligand protein-1 |
| PDX | Patient-derived xenograft |
| pHGG | Pediatric High Grade Glioma |
| PPN | Pedunculopontine nucleus |
| PRC2 | Polycomb repressive complex 2 |
| R/R | Relapsed/Refractory |
| Rb | Retinoblastoma tumor suppressor |
| RMAT | Regenerative Medicine Advanced Therapy |
| scRNA-seq | single-cell RNA sequencing |
| SIRPa | CD47-Signal retention protein alpha |
| TAA | Tumor-associated antigen |
| TCR | T cell receptor |
| TGF-β | Transforming growth factor β |
| TIAN | Tumor inflammation-related neurotoxicity |
| TIM-3 | T cell immunoglobulin and mucin domain-containing protein 3 |
| TIS | Tumor inflammation signature |
| TMB | Tumor mutational burden |
| TME/TIME | Tumor (Immune) microenvironment |
| TSA | Tumor-specific antigen |
| TSP1/Thbs1 | Tumor-derived thrombospondin 1 |
| WHO | World Health Organization |
| WT | Wild type |
